# Integration of Sensory and Reward Information during Perceptual Decision-Making in Lateral Intraparietal Cortex (LIP) of the Macaque Monkey

**DOI:** 10.1371/journal.pone.0009308

**Published:** 2010-02-19

**Authors:** Alan E. Rorie, Juan Gao, James L. McClelland, William T. Newsome

**Affiliations:** 1 Howard Hughes Medical Institute and Department of Neurobiology, Stanford University School of Medicine, Stanford, California, United States of America; 2 Department of Psychology, Stanford University, Stanford, California, United States of America; Mount Sinai School of Medicine, United States of America

## Abstract

Single neurons in cortical area LIP are known to carry information relevant to both sensory and value-based decisions that are reported by eye movements. It is not known, however, how sensory and value information are combined in LIP when individual decisions must be based on a combination of these variables. To investigate this issue, we conducted behavioral and electrophysiological experiments in rhesus monkeys during performance of a two-alternative, forced-choice discrimination of motion direction (sensory component). Monkeys reported each decision by making an eye movement to one of two visual targets associated with the two possible directions of motion. We introduced choice biases to the monkeys' decision process (value component) by randomly interleaving balanced reward conditions (equal reward value for the two choices) with unbalanced conditions (one alternative worth twice as much as the other). The monkeys' behavior, as well as that of most LIP neurons, reflected the influence of all relevant variables: the strength of the sensory information, the value of the target in the neuron's response field, and the value of the target outside the response field. Overall, detailed analysis and computer simulation reveal that our data are consistent with a two-stage drift diffusion model proposed by Diederich and Bussmeyer [1] for the effect of payoffs in the context of sensory discrimination tasks. Initial processing of payoff information strongly influences the *starting point* for the accumulation of sensory evidence, while exerting little if any effect on the *rate* of accumulation of sensory evidence.

## Introduction

One of the most successful enterprises of experimental psychology and systems neuroscience has been the elucidation of mechanisms underlying simple forms of decision-making. Green and Swets [2] provided the theoretical groundwork for this effort with their theory of signal detection. Incorporating Bayesian principles, Green and Swets accounted for the psychophysical decisions of human subjects in numerous circumstances by invoking an optimal combination of sensory information about the stimulus and prior information about the probability of a particular response being correct. The final weight of evidence favoring one or the other response was expressed as the “likelihood ratio”, a formulation that has exerted a prodigious impact on subsequent studies of decision-making and on the development of artificial decision-making algorithms.

While the original formulation of Green and Swets was designed only to account for the *accuracy* of choice data, a rich body of experimental and theoretical work subsequently extended the insights of signal detection theory into dynamical models in which evidence is accumulated gradually over time during single trials. Originating in seminal work by Laming [3], Link and Heath [4] and Ratcliff [5], these models depicted the decision mechanism as a “diffusion” process in which a decision variable assumes a neutral value at the beginning of a trial, then “drifts” gradually under the influence of incoming sensory information toward a “barrier”. The decision is reached when the diffusing decision variable encounters the specified barrier, or threshold. The key variables in such models are the starting point of the diffusion process, the drift rate of the decision variable under the influence of incoming sensory information, the distance between the starting point and the decision barrier, and noise associated with all three variables. A related class of models, called accumulator models, invokes separate accumulators to model forced-choice tasks with two or more alternatives [6], [7], and recent versions of such models allow for the possibility of competition among the accumulators and decay or leakage of accumulated information (e.g. [8], [9], [10]). These models generate remarkably precise fits to both accuracy and reaction time data with relatively few parameters, and simulations have demonstrated the feasibility of implementing the models in recurrent networks of biophysically realistic neurons [11], [12], [13], [14], [15]. As Ratcliff and McKoon [16] have recently observed: “It has probably not been realized in the wider scientific community that the class of diffusion models has as near to provided a solution to simple decision making as is possible in behavioral science.”

Given the success of diffusion models in accounting for large classes of behavioral data, neurophysiologists have naturally employed these models to examine the neural mechanisms underlying simple forms of decision-making. Hanes and Schall [17] showed that a diffusion model accounts well for variability in saccadic reaction times measured in monkeys performing a countermanding task, and more impressively, they demonstrated that the underlying signals measured from single neurons in the frontal eye field are well described by the drift rate variable, but poorly described by the threshold variable, of an underlying diffusion process. This initial finding led to a substantial body of work suggesting that diffusion models account well for the neural mechanisms underlying saccade generation in several contexts [18].

Shadlen and colleagues opened a particularly rich vein of research by applying accumulator models to study neural mechanisms underlying the workhorse task of psychophysics—the two-alternative, forced-choice (2AFC) sensory discrimination. Using a discrimination of motion direction in which monkeys indicate their decisions by making saccadic eye movements [19], [20], Shadlen and colleagues showed that a competing accumulator model can account well for behavioral accuracy and reaction time data, and they demonstrated that the dynamical activity of single neurons in the lateral intraparietal area of monkey cortex (LIP) is also well described by a noisy information accumulation process [21], [22], [23], [24], [25]. Gold and Shadlen [21], [26] further suggested that LIP neurons combine accumulated sensory information with additional sources of information (e.g. prior probability and payoffs) to form decision variables that are monotonically related to the log of the likelihood ratio of choosing one alternative versus the other, and are thus ideally suited for guiding decisions about where to move the eyes. These models postulate that separate populations of neurons in LIP correspond to accumulators that encode a quantity proportional to the log likelihood ratio associated with each of the two alternatives. Hanks, Dietterich and Shadlen [27] subsequently obtained microstimulation data consistent with the idea that LIP activity is not merely correlative, but plays a causal role in these decisions. Importantly, Ratcliff and colleagues have shown that “build-up” neurons in the superior colliculus exhibit very similar properties during a 2AFC discrimination, raising the possibility that accumulation of information into a decision variable is accomplished by a distributed network of neural circuits within oculomotor planning structures [28], [29].

These findings raised the intriguing question of whether the role of LIP in decision-making is specific to the accumulation of sensory information, or whether, in the spirit of signal detection theory, LIP incorporates a broader range of inputs known to influence behavioral decisions. The answer to this question has proven to be emphatically affirmative. LIP activity is now known to reflect numerous variables relevant to behavioral decisions, including the prior probability that a particular eye movement will be instructed [30], the probability of obtaining a reward during foraging or competitive games [30], [31], [32], [33], [34], addition and subtraction of probabilistic quantities [35], [36], the internal confidence associated with a sensory decision [36], and—remarkably—the social value of ethologically powerful stimuli which can override the intrinsic appeal of liquid rewards to a thirsty animal [37].

Somewhat surprisingly, the emerging model of LIP computation has not yet been tested by manipulating payoffs in the context of a sensory discrimination task. Payoffs, like prior probabilities, are known to bias choices in near-threshold discrimination tasks, and this effect can be easily incorporated into the likelihood ratio to create a payoff-weighted likelihood function [2], [21], [26]. Furthermore, the effects of payoff information on discrimination accuracy and reaction times are well described by drift diffusion models that postulate a two-stage accumulation process—an initial stage of accumulation about the payoffs followed by a second stage of accumulation of sensory information [1], [38], [39]. In the two-stage model, the initial accumulation of payoff information sets the starting point of the diffusion process that accumulates sensory information, a postulate that is well supported by fits to the behavioral data. In contrast to the strong effect of payoff information on starting point, fits to the behavioral data indicated that payoff information had little or no effect on the drift rate of the diffusion process.

Our primary goal in this paper is to examine the effect of explicit payoff information on the activity of LIP neurons while monkeys perform a 2AFC perceptual discrimination task. It is known that LIP neurons are sensitive to the magnitude of a reward associated with a visual cue or an eye movement target [30], [40], [41], but it is not known how LIP processes sensory and reward signals when the animal must balance the two (sometimes conflicting) sources of information in making decisions. We therefore trained two rhesus monkeys to perform the classic random dot motion discrimination task in which the perceived direction of motion is indicated by a saccadic eye movement to one of two visual targets corresponding to the two possible directions of coherent motion [19], [20]. The important modification was that the size of the reward for a correct response to each of the two possible directions of motion was manipulated (single vs. double reward). Reward magnitude for each alternative was cued in advance by the color of the saccade target corresponding to each possible choice. Unequal rewards led to a choice bias in favor of the more highly rewarded target, and analysis of the behavioral data demonstrated that the induced choice bias was nearly optimal for maximizing overall reward rate [42].

To determine how sensory and reward information are integrated at the cellular level, we recorded from single neurons in LIP while the monkeys performed this task. Our analyses were designed to address four questions, the first two descriptive and last two mechanistic: 1) Are reward and sensory information integrated at the level of single neurons, and if so, in what proportions and with what dynamics? 2) Are individual LIP neurons influenced only by the reward value of the target in the response field, or do they also reflect the reward associated with the alternative target, so that they can reflect relative as well as absolute reward magnitude [32], [34], [35]? 3) Are the dynamics of LIP activity consistent with a two-stage diffusion model as proposed by Diederich and Bussmeyer [1]? 4) Does payoff information affect the *starting point* of the sensory accumulation process in LIP as suggested by the model of Diederich and Bussmeyer, or does its influence accumulate gradually along with the accumulation of sensory information (drift rate)? Our data address each of these questions, thereby shedding new light on the neural basis of oculomotor decisions and the relationship of neural activity to formal models of decision-making.

## Methods

### Subjects and Ethics Statement

Two adult male rhesus monkeys, A and T (12 and 14 kg), were trained on a two-alternative, forced-choice, motion discrimination task with multiple reward contingencies. Daily access to fluids was controlled during training and experimental periods to promote behavioral motivation. Before training, the monkeys were prepared surgically with a head-holding device [43] and a scleral search coil for monitoring eye position [44]. All surgical, behavioral, and animal care procedures complied with National Institutes of Health guidelines and were approved by the Stanford University Institutional Animal Care and Use Committee. Ethical standards incorporated into these guidelines and into our routine laboratory procedures include a psychological enrichment program, frequent contact with other animals (visual, auditory, olfactory and, where appropriate, touch and grooming), regular veterinary supervision and care, and pharmacological amelioration of pain associated with surgeries.

### A Motion Discrimination Task with Multiple Reward Contingencies

On each behavioral trial the monkeys observed a noisy random-dot motion stimulus and reported which of two possible directions of motion were present by making a saccadic eye movement to one of two targets. The motion stimulus was composed of dynamic random dots, viewed through a circular aperture on a dark computer screen. On each trial a variable proportion of the dots moved coherently in one of two opposite directions while the remaining dots were flashed transiently at random locations and times (for a detailed description see [45], [46], [47]). The difficulty of the discrimination was varied parametrically from trial-to-trial by adjusting the percentage of dots in coherent motion: the task was easy if most of the dots moved coherently (e.g. 50% or 100% coherence), but became progressively more difficult as the coherence decreased.

Importantly, the coherence only describes the strength of the motion, not its direction. In the data figures that follow, the “sign” of the coherence indicates the direction of coherent motion. Thus +25% coherence and –25% coherence are equally strong motion signals, but move in opposite directions. Typically, the animals viewed a range of signed coherences spanning psychophysical threshold. The animals were always rewarded for indicating the correct direction of motion, except at 0% coherence where they were rewarded randomly (50% probability) irrespective of their choice.

**Figure 1:**
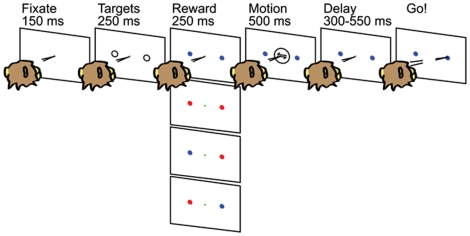
A two-alternative, forced-choice, motion discrimination task with multiple reward contingencies; sequence of events comprising a typical trial. From left to right, trials begin with the onset of a fixation point. Two saccade targets appear and then change color indicating the magnitude of the reward available for correctly choosing that target. A blue target indicates a low magnitude (L) reward, while a red target indicates a high magnitude (H) reward. The four reward conditions are depicted vertically—LL,HH, LH and HL, from top to bottom. The visual motion stimulus is centered on the fixation point. Following offset of the motion stimulus, the monkey maintains fixation during a variable delay period after which the fixation point disappears, cueing the monkey to report his decision with a saccade to the target corresponding to the perceived direction of motion. If the monkey chooses the correct direction of motion, he receives the reward indicated by the color of the chosen target.

Figure 1 illustrates the sequence of events comprising a typical trial of the motion discrimination task. From left to right, trials began with the onset of a small dot that the monkey was required to fixate for 150 ms. Next, two saccade targets (hollow gray circles) appeared for 250 ms. The two targets were 10 degrees eccentric from the visual fixation point and 180 degrees apart from each other. The targets were positioned in-line with the axis of motion being discriminated. By convention, the target corresponding to positive coherence is target 1 (T1) while the other is target 2 (T2). Target 1 was placed in the response field of the LIP neuron under study (see below), while target 2 was placed in the opposite hemifield.

After 250 ms the targets changed color, indicating the magnitude of reward available to the monkey for correctly choosing that target. A blue target indicated a low magnitude (L) reward (1 unit, ∼0.12 ml of juice), while a red target indicated a high magnitude (H) reward (2 units). As there are two reward magnitudes (H and L) to be assigned to each of two target locations (T1 and T2), there were four reward conditions overall, schematized by the vertical row of panels in Figure 1: 1) the LL condition in which both targets were blue, 2) the HH condition in which both targets were red, 3) the HL condition, in which T1 was red and T2 was blue, and 4) the LH condition which was the mirror of the HL condition.

The colored targets were visible for 250 ms before onset of the visual motion stimulus, which appeared for 500 ms, centered on the fixation point. Following offset of the motion stimulus, the monkey was required to maintain fixation for a variable delay period (300–550 ms) after which the fixation point disappeared, cueing the monkey to report his decision with a saccade to the target corresponding to the perceived direction of motion. If the monkey chose the correct direction of motion, he received the reward indicated by the color of the chosen target.

Fixation was enforced throughout the trial by requiring the monkey to maintain its eye position within an electronic window (1.25 degrees radius) centered on the fixation point. Inappropriate breaks of fixation were punished by aborting the trial and enforcing a time-out period before onset of the following trial. Psychophysical decisions were identified by detecting the time of arrival of the monkeys' eye in one of two electronic windows (1.25 radius) centered on the two choice targets (T1 and T2).

All trials were presented pseudo-randomly in block-randomized order. For monkey A, we employed 12 signed coherences, 0% coherence and four reward conditions, yielding 52 conditions overall. For monkey T we eliminated four of the lowest motion coherences because this animal's sensitivity to the motion stimulus was somewhat lower than monkey A's. Thus monkey T was tested for 36 conditions overall. We attempted to acquire 40 trials for each condition, enabling us to characterize a full psychometric function for each of the four reward conditions. Because these behavioral data were obtained simultaneously with electrophysiological recordings, however, we did not always acquire the full 40 trials for each condition (the experiment typically ended when single unit isolation was lost). For the data reported in this paper, the number of repetitions obtained for each experiment ranged from 8 to 40 with a mean of 36.

The full data set analyzed in this paper consists of 33 behavioral sessions from monkey A and 24 sessions from monkey T. Multiple LIP neurons were sometimes recorded simultaneously—either from multiple electrodes or a single electrode (see below)—yielding a total of 51 LIP neurons from monkey A, and 31 from monkey T.

### Procedures

During both training and experimental sessions monkeys sat in a primate chair at a viewing distance of 57 cm from a color monitor. Visual stimuli were presented on the monitor under computer control. The monkeys' heads were positioned stably using the head-holding device, and eye position was monitored throughout all experimental sessions by means of a magnetic search coil apparatus (0.1° resolution; CNC Engineering, Seattle, WA).

Area LIP was identified by a combination of stereotactic location, characteristic physiological activity and anatomical magnetic resonance imaging. Single neurons were isolated and their activity recorded with extracellular microelectrodes. Monkey T received a single craniotomy that matched the dimensions of the recording cylinder. For monkey A, the cylinder was placed on intact skull protected with a thin layer of dental acrylic. For this animal, a 3 mm “burr-hole” was drilled, under surgical conditions, one day before beginning recordings at a given location within the recording cylinder.

For monkey A, neurophysiological recording was accomplished with quartz/platinum-tungsten electrodes (Thomas Recording, Giessen, Germany) that were positioned and manipulated daily with a 5-channel single electrode system (“Mini Matrix,” Thomas Recording, Giessen, Germany). Recordings were typically made with two to four electrodes. For monkey T, we employed tungsten electrodes (FHC Inc., Bowdoin, Maine) positioned with a Crist grid (Crist Instruments Co., Inc., Hagerstown, Maryland) and manipulated with a Narishige single electrode drive (Narishige Co., LTD, East Meadow, New York). Multiple neurons were recorded simultaneously in 13 experiments for monkey A, and in 6 experiments for monkey T. In these experiments target 1 was positioned so as to activate all response fields (simultaneous recordings were only carried out if the response fields overlapped substantially). When multiple neurons were recorded, spikes were sorted and clustered off-line, based on a principle component analysis of the resulting voltage waveforms using the Plexon off-line sorter (Plexon Inc., Dallas, Texas).

Behavioral control and data acquisition were managed by a PC-compatible computer running the REX software environment [48] and QNX Software System's (Ottawa, Canada) real-time operating system. Visual stimuli were generated using a VSG graphics card (Cambridge Graphics, UK) and presented on a CRT display. After amplification, single unit spiking activity was identified and collected along with digitized task events and eye position traces using the Plexon (Plexon Inc., Dallas, Texas) data acquisition system operating in conjunction with Rex. All data were subsequently analyzed offline with custom scripts written in the MATLAB (The MathWorks, Inc., Natick, Massachusetts) programming language, running on Apple computers (Apple Computer, Inc., Cupertino, California).

### Cell Selection

We selected for study LIP neurons that exhibited persistent delay-period activity during a delayed saccade task. Informal observations in prior studies indicate a strong correlation between this property and “choice predictive” activity in the motion discrimination task [49], [50]. Neurons with persistent activity in the delayed saccade task comprised roughly a third of all LIP neurons encountered. We employed a variant of the delayed saccade task that has been used extensively to identify these neurons. Each trial began with the onset of a small fixation target. After the monkey acquired and fixated the target for 150 ms, a single saccade target appeared for a variable delay period (250–800 ms). At the end of the delay period the fixation point disappeared, cueing the monkey to saccade to the target. Completed trials were identified by detecting the time of arrival of the monkey's eye in an electronic window (1.25 radius) centered on the target. The saccade target was typically presented in pseudorandom order at six locations—10 degrees eccentric and separated by equal polar angles. Eccentricities and angles were sometimes varied to locate the sensitive region of a given neuron's RF.

### Analysis of Psychophysical Data

We fitted psychophysical data with a logistic regression model that describes the log-odds-ratio of choosing T1 as a function of the linear sum of signed coherence and the values of each of the two targets:

Equation 1:
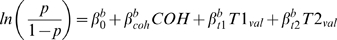
Where p is the observed probability of choosing T1, and β_coh_, β_t1_ and β_t2_ are the fit coefficients representing the effects of motion coherence and target values on this probability (the superscripts, *b*, indicate coefficients for behavioral data, as opposed to physiological data in Equation 3 below). β_0_ represents any global bias the monkey has towards choosing T1. COH is the coherence of the motion stimulus, in fractional units of the maximum coherence employed and signed to signify the direction as described above. Thus, COH ranges from -1 to 1, where -1 represents −48% coherence and +1 represents +48% coherence. T1_val_ and T2_val_ are assigned either +1, if the target was H, or −1 if the target was L. For example, on HL trials in which the motion coherence was −12%, COH  = −0.25, T1_val_  = +1 and T2_val_  = −1. Constraining these factors to be in the same range (−1 to 1) allows us to compare directly the values of the fit coefficients.

**Figure 2:**
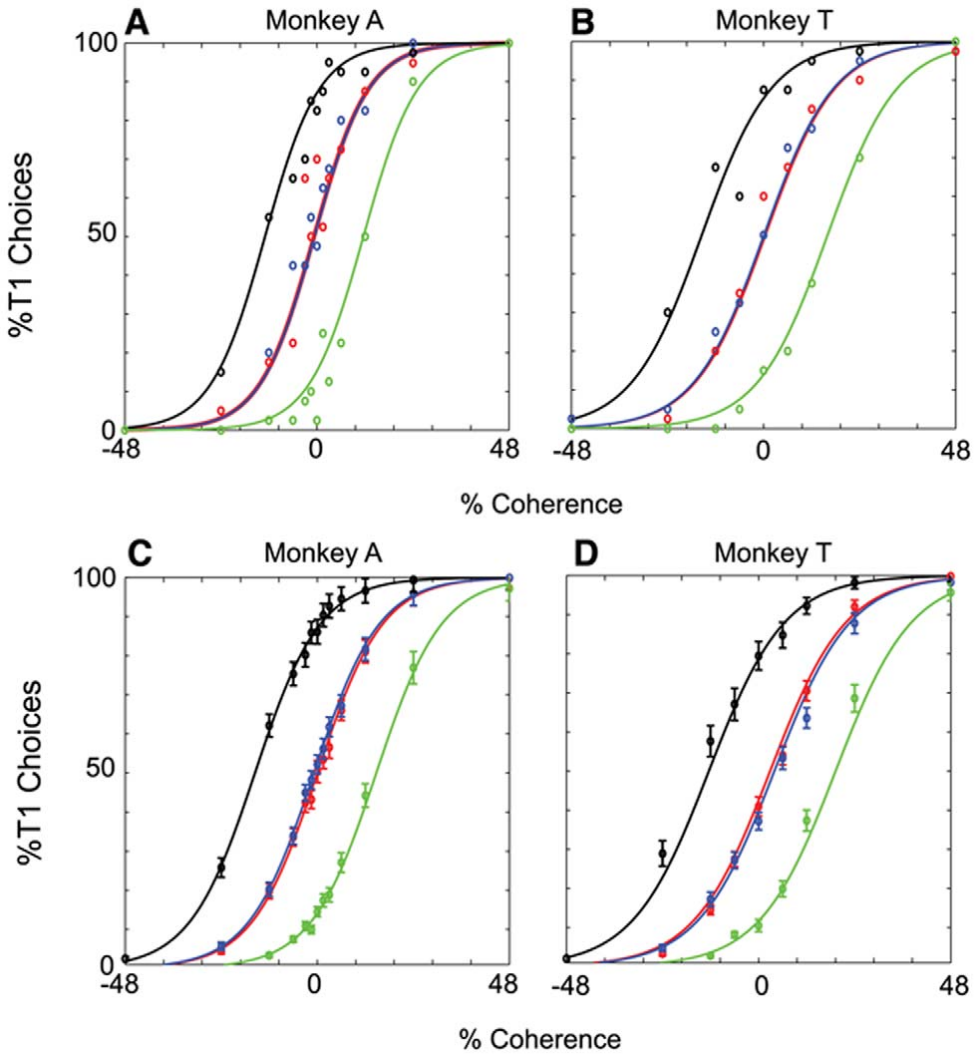
Relative reward biases choice. **A–D**. Psychometric functions (PMFs) describing each monkey's probability of choosing T1 as a function of motion coherence. Motion coherence is denoted with a magnitude indicating the strength of the motion and a sign indicating its direction. Positive coherence denotes motion towards T1 while negative coherence denotes motion towards T2. Separate PMFs are plotted for each reward condition (HH, red; LL, blue; HL, black; LH, green). Circles depict the observed proportion of T1 choices, and sigmoidal curves are fit quantitatively with logistic regression. **A–B**. Results from one representative experiment for monkey A and monkey T, respectively. **C–D**. Average PMFs across all behavioral sessions for monkeys A (n = 33) and T (n = 24), respectively.

Equation 1 can be rearranged to Equation 2, which was used to generate the sigmoid functions in Figure 2.

Equation 2:
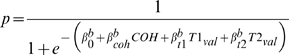



### Analysis of electrophysiological data

For each neuron, electrophysiological data were analyzed by means of a multi-variable, linear regression model:

Equation 3:
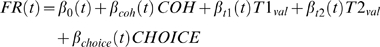
where FR(t) is the mean firing rate over a given temporal epoch and trial, and β_coh_, β_t1_ and β_t2_ and β_choice_ are fit coefficients representing the effects of motion coherence, target values and choice on firing rate. As with the analysis of psychophysical data, COH is the coherence of the motion stimulus on that trial, in fractional units of the maximum coherence employed and signed to signify the direction of motion. Similarly, T1_val_ and T2_val_ are assigned either +1, if the target was H, or −1 if the target was L. Choice is assigned a value of +1 for T1 choices and −1 for T2 choices. As with the psychophysical analysis, constraining these factors to be in the same range (−1 to 1) allows us to compare directly the values of the fit coefficients and determine which have greater impact on FR. Note that Equation 3 is very similar to Equation 1 with the addition of a factor for behavioral choice. Equation 3 was fit to the average firing rate for each neuron in sliding 50 msec time windows.

To ensure that the effects on firing rate of coherence, target value and choice (equation 3) did not arise artifactually from subtle variations in the operant saccades, we also fit all data with a regression model that included several saccade parameters as coregressors:

Equation 4:
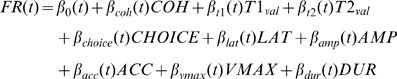
The latency, amplitude, accuracy, maximum speed and duration of the saccade are represented respectively by LAT, AMP, ACC, VMAX and DUR.

**Figure 3:**
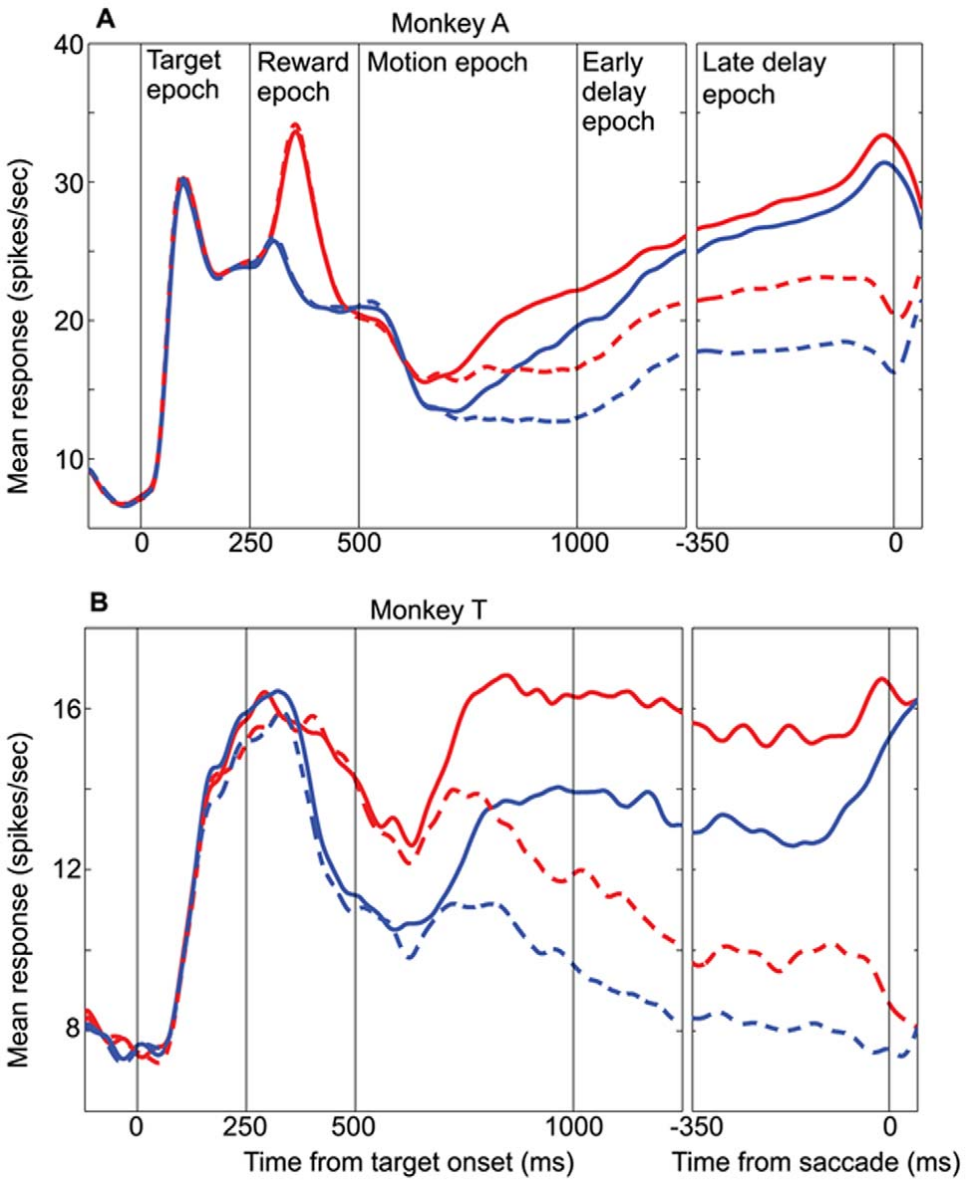
LIP represents the absolute value of the option in the RF. **A.** Average data from monkey A (n = 51 cells). **B.** Average data from monkey T (n = 31 cells). Mean LIP firing rate as a function of time, for the HH (red) and LL (blue) reward conditions. Data are plotted separately for T1 (solid) and T2 (dashed) choices. 0–250 ms is the target epoch in which the blank targets are presented; 250–500 ms is the reward epoch in which the targets change color to cue the reward condition; 500–1000 ms is the motion epoch in which the random-dot motion stimulus is presented; 1000–1250 ms is the early segment of the delay epoch; −350–0 ms (in the right panel) is the late delay epoch immediately preceding the saccade. Any difference between the red and blue curves indicates an effect of the absolute value of the option in the RF.

To evaluate possible neural mechanisms underlying the accumulation of sensory information by LIP neurons, we assessed the rate of the increase in neural activity during the motion epoch for each LIP neuron, generally following the procedure of Kiani and Shadlen [36]. For each neuron, we first identified the well known “dip” in LIP activity that follows the onset of motion stimulus (see Figs. 3–5, first half of the motion epoch). We calculated the mean peristimulus-time histogram (PSTH) of neural activity for all correct T1 choices and considered the “dip” to be the point of minimum activity between 50 ms and 300 ms after stimulus onset. After identifying the time of the dip, we then calculated for each reward condition the average rate of rise (or fall) of neural activity between the dip and 1) the end of the motion epoch, or 2) the point at which average firing rate saturated—whichever came first. For neurons whose firing rate saturated before the end of the motion epoch, we considered the point of saturation to be the time at which the maximum firing rate was achieved. We define the slope of the accumulation process to be the best linear fit to the PSTH during this measurement window. To obtain temporally independent data for this analysis, the average firing rate during the measurement window was calculated in 50 msec bins with no overlap.

**Figure 4 pone-0009308-g004:**
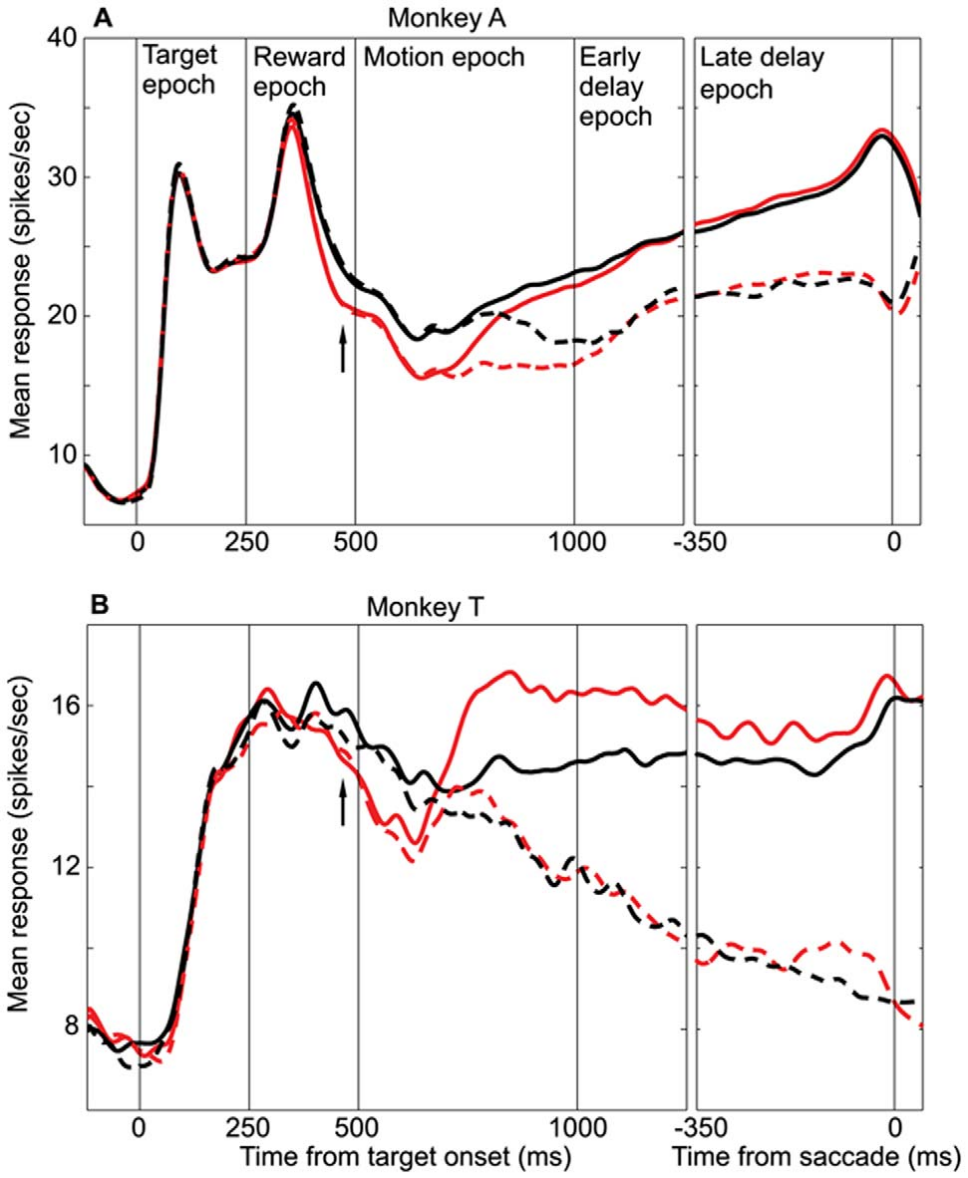
LIP represents the relative value of the option in the RF. **A.** Average data from monkey A (n = 51 cells). **B.** Average data from monkey T (n = 31 cells). Mean LIP firing rate as a function of time, for the HH (red) and HL (black) reward conditions. HH curves are the same as in Figure 3A–B. Data are plotted separately for T1 (solid) and T2 (dashed) choices. In the left panels, responses are aligned to the target onset, while in the right panels, responses are aligned to the saccade time. Any difference between the red and black curves indicates an effect of the relative value of the option in the RF.

**Figure 5 pone-0009308-g005:**
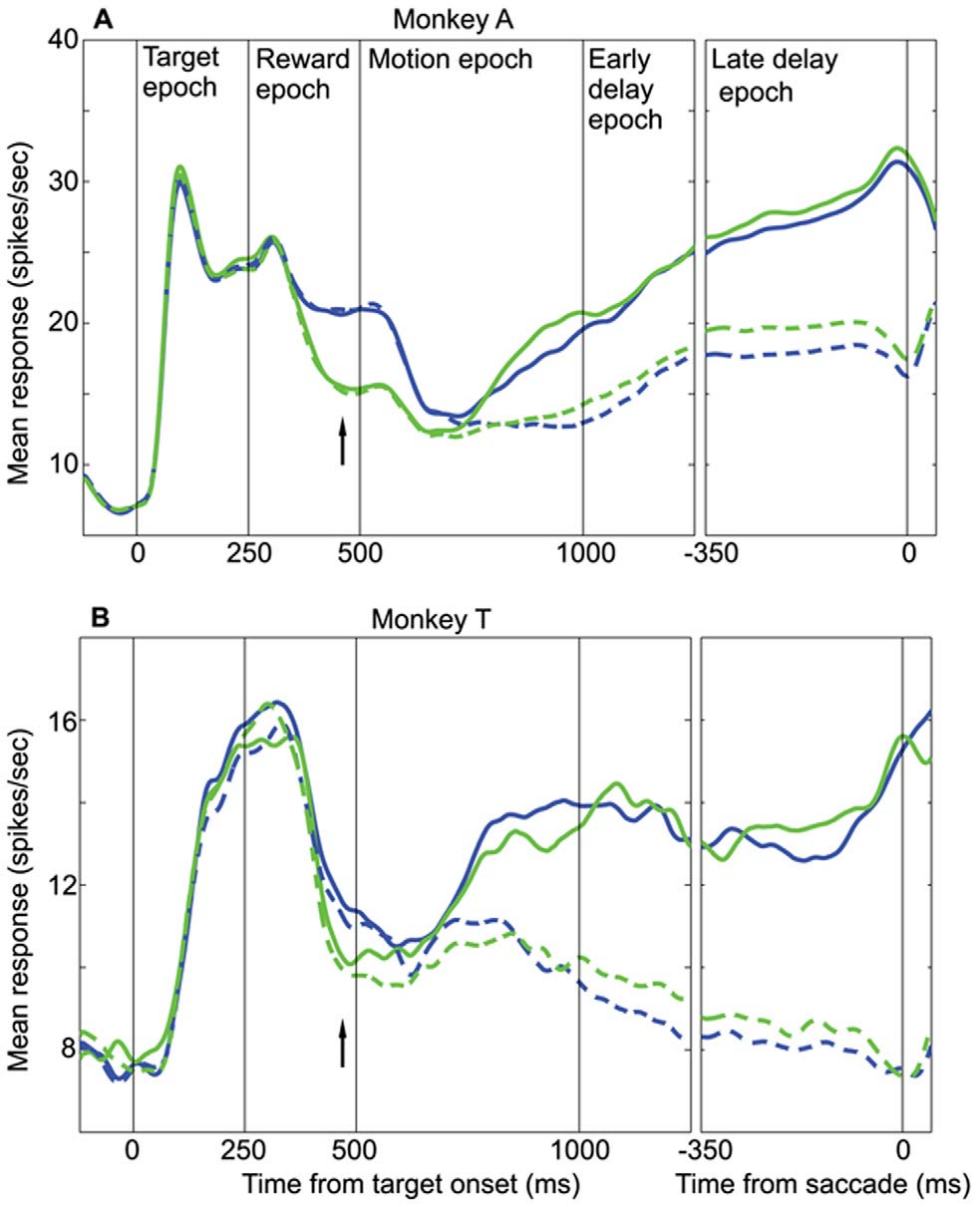
A second look at the relative value effect. **A.** Average data from monkey A (n = 51 cells). **B.** Average data from monkey T (n = 31 cells). Mean LIP firing rate as a function of time, for the LL (blue) and LH (green) reward conditions. LL curves are the same as in Figure 3A–B. Data are plotted separately for T1 (solid) and T2 (dashed) choices. In the left panels, responses are aligned to the target onset, while in the right panels, responses are aligned to the saccade time. Any difference between the blue and green curves indicates an effect of the relative value of the option in the RF.

The central goal of the slope calculation was to compare the average rate of rise of LIP firing rates for the four reward conditions, while factoring out any contribution of behavioral choice or the motion stimulus itself (signed coherence). We eliminated possible effects of choice by analyzing only trials that ended in T1 choices, as indicated above. We neutralized the effects of stimulus strength by conducting the analysis in the following steps. 1) For each trial, we offset the ‘firing rate vs. time’ trace so that the firing rate was zero at the dip. Thus all firing rates during the measurement window (defined above) are calculated relative to the firing rate at the dip. 2) For each neuron, for all trials at a given signed coherence level, the firing rate in each 50 msec bin was normalized to the maximum firing rate (relative to the dip) observed in any single bin on any trial during the measurement window. 3) For each signed coherence level, normalized firing rates were averaged within time bins across trials. Averages were calculated separately for the four reward conditions. 4) For each reward condition, normalized firing rates were averaged across all positive coherence levels to obtain, for each neuron, a normalized, reward-condition-specific PSTH. (T1 choices were relatively rare at negative coherences, and we therefore omitted negative coherences from the average to avoid measurement noise resulting from small numbers of trials.) 5) For each neuron, the slope of the PSTH was measured for each reward condition during the measurement window. 6) Statistical tests were performed to identify differences in slopes between reward conditions across the population (see Discussion). 7) For each reward condition, normalized firing rates were then averaged across neurons to obtain an average PSTH for the entire population of neurons for each animal.

## Results

### “Relative Value” Biases Choice

Figures 2A–D illustrate psychometric functions (PMFs) depicting the observed proportion of T1 choices as a function of signed motion coherence. A separate PMF is plotted for each of the four reward conditions: high-high (HH; large rewards available for both targets), low-low (LL; small rewards available for both targets), high-low (HL; large reward for target 1 and small reward for target 2) and low-high (LH; vice-versa) (see Methods). The sigmoidal curves are logistic regression fits to the observed data (Methods). Figures 2A and 2B depict data from a representative experiment for monkey A and monkey T respectively. Figures 2C and 2D depict the average PMF across all behavioral sessions for monkeys A (n = 33) and T (n = 24) respectively.

Two features of the data in Figure 2 are notable. First, the PMFs for the unbalanced reward conditions are shifted horizontally with respect to the balanced conditions, revealing a systematic choice bias for the larger reward. Both monkeys chose T1 more frequently when it was associated with a high reward relative to T2 (black symbols and lines), and chose T2 more frequently for the converse condition (green symbols and lines). Second, the observed behavior for the balanced conditions (HH and LL reward conditions—red and blue circles, respectively) is nearly identical, indicating that the monkey's probability of choosing T1 is unaffected by changes in the absolute size of the reward. One might have expected the PMFs to steepen for the HH condition if the monkeys were motivated by the larger rewards to discriminate the motion stimulus more carefully. Instead, both monkeys appear to discriminate as well as they possibly can for both conditions, suggesting a high baseline level of motivation throughout the experiments. Both monkeys, however, were significantly more likely to break fixation during LL trials as compared to HH trials (LL trials: monkey A, 2.73%+/−0.13; monkey T, 3.19+/−0.35; HH trials: monkey A, 1.77%+/−0.12; monkey T, 1.66%+/−0.27; two-sample t-test, monkey A, p<10∧-4, monkey T, p<0.002).

For both monkeys, average behavior across all experiment sessions (Fig. 2C,D) was very similar to the individual session examples (Fig. 2A,B). Thus the effects of coherence and reward size on psychophysical choices were robust and consistent within and across the data sets for the two monkeys. As we have reported previously [42], the observed choice biases are nearly optimal in terms of maximizing reward collection across an experimental session. On average, both monkeys harvested rewards at ∼98% of the theoretical maximum given their underlying psychophysical sensitivity to the motion stimulus.

### The Representation of Choice, Absolute Value, and Relative Value in LIP

Our analysis of LIP activity during this task revealed three primary effects that varied dynamically over the course of a typical trial: 1) the well known effect of decision outcome (choice), particularly in the later stages of the trial, 2) an effect of the “absolute value” of the target in the neuron's RF (T1) irrespective of the value of T2, and 3) an effect of the “relative value” of the target in the neuron's RF (whether it was larger than, smaller than, or equal to the value of T2). In the following three sections, we will illustrate each of these effects and its dynamics qualitatively by inspection of average PSTH's for each monkey. In the fourth section we will analyze these effects quantitatively by means of a multiple regression analysis.

As described in Methods, we always positioned one response target (T1) within the RF of the neuron under study, while positioning the other target (T2) 180° away in the opposite hemifield. The axis of stimulus motion was defined by these two target positions so that motion discrimination choices corresponded to saccades into or out of the RF. In the following sections, we denote choices into the RF as “T1 choices” and those to the opposite target as “T2 choices”.

### Representation of Choice: Qualitative Description

Figures 3A (monkey A) and 3B (monkey T) depict mean LIP firing rate as a function of time for all successfully completed trials in the HH (red) and LL (blue) reward conditions. Data are plotted separately for trials in which the monkey chose T1 (solid lines) and T2 (dashed lines). Both 3A and 3B consist of two panels: a left panel with responses aligned to the time of target onset and a right panel (labeled “late delay epoch”) with responses aligned to the time of the saccade. The black vertical lines in both figures denote relevant task epochs.

Note first that in both 3A and 3B, the solid and dashed lines are initially identical (for each color), but diverge approximately 200 ms into the motion period. Thus, shortly after the onset of the motion stimulus, LIP neurons in both monkeys begin to signal choice—whether the monkey will choose T1 or T2. This result is not surprising. We explicitly selected for study neurons that responded differentially to oppositely directed eye movements in the delayed saccade task, and it is well known from previous work that such LIP neurons typically exhibit “choice predictive” activity during a variety of forced-choice tasks [30], [49], [51]. The data in Figure 3 demonstrate that this property of LIP neurons holds for a task in which decisions are based on a combination of visual motion and reward information. The effect of behavioral choice in the LIP data is robust, consistent across neurons and monkeys, and present for all four reward conditions as demonstrated below.

An unanticipated difference between the two monkeys was the absence of an initial visual “burst” in monkey T. The burst was absent during the delayed saccade task as well (data not shown). Although LIP neurons lacking the visual burst have been observed by our lab and others previously, we have never recorded from a monkey in which the burst appeared to be absent across the population. We do not believe this result is due to oversampling from a few unusual locations in LIP; our recordings sites were reasonably widely distributed along the lateral bank of the intraparietal sulcus. While this appears to be a genuine difference between the monkeys, it does not affect any of our key results pertaining to the accumulation of motion information or the influence of reward condition on LIP activity since these results are present in both monkeys.

### Representation of Absolute Value: Qualitative Description

Any differences in neural activity between the HH and LL conditions indicate an effect of absolute reward value since the relative reward value (compared to the value of T2) is identical in the two conditions. By comparing the red and blue lines in Figure 3 we can see the extent to which LIP represents absolute reward value. Consider first the data from monkey A in Figure 3A. The solid red and blue traces (T1 choices) separate with very short latency following presentation of the reward cues at 250 ms. Thus the LIP population rapidly encodes the absolute value of T1, producing elevated firing rates when a high value target is presented within the RF. Following their initial separation, the red and blue traces converge briefly near the beginning of the motion epoch, but then separate again for the duration of the trial. Qualitatively, then, except for a brief interval near the onset of the motion stimulus, LIP neurons from monkey A encode a signal concerning the absolute value of the reward available in the RF throughout the trial. Note that this representation of absolute value is present for T2 choices as well (dashed traces).

Figure 3B shows a similar pattern of activity for the LIP population recorded from monkey T. Even though LIP activity in monkey T does not respond as rapidly or robustly as in monkey A, all major features of the absolute value signal observed in monkey A are replicated in monkey T: 1) the effect of absolute value begins during the reward cue period, 2) greater absolute value is represented by higher firing rates, 3) the effect is maintained until the end of the trial and 4) the effect is present for T2 choice trials as well. A minor difference is that the absolute reward signal does not “disappear” at any point in the trial for monkey T. It is interesting that absolute reward value exerts a substantial effect on LIP activity even though it exerts little if any effect on choice (Fig. 2). We will consider this point further in the simulations and in the Discussion.

### Representation of Relative Value: Qualitative Description

As revealed by the behavioral data, the relative reward value of the two targets exerts a substantial impact on choice behavior. We first examine the effect of relative value on LIP by comparing neuronal responses in the HH and HL reward conditions. In these conditions, the value of T1 is constant (high value) while the value of T2 differs (high in HH, low in HL). Thus, any LIP modulation between these two conditions indicates a relative effect of T2 value on the response to the high value target present in the RF. Figures 4A and 4B depict LIP responses for monkeys A and T, respectively, to the HH (red traces) and HL (black traces) reward conditions. The format of these figures is identical to Figures 3A and 3B, and the red curves are the same as in Figure 3.

In Figure 4A, the black and red traces separate late in the reward cue epoch, with the average firing rate being higher for the HL condition (arrow). This difference indicates that on average, LIP neurons respond more strongly to a target in the RF (T1) when it has a larger value *relative* to that of the T2 target. This “relative value” signal is present throughout most of the motion epoch but disappears early in the delay epoch, after the choice has presumably been determined. The same dynamics are evident both for T1 and T2 choices (solid and dashed lines, respectively).

A similar pattern of activity is present for the population data from monkey T, illustrated in Figure 4B. As for monkey A, the relative reward signal emerges late in the reward cue epoch (black arrow), with average firing rate being higher for larger relative value. For monkey T, however, the relative reward signal fades more rapidly than for monkey A. Additionally, for T1 choices, the relative reward signal inverts during the second half of the motion epoch and remains inverted throughout the delay epoch. This inversion is not present for T2 choices, however.

We can acquire a second look at the effects of relative reward by comparing the LL and LH reward conditions. As in the previous comparison of HH and HL trials, the value of T1 is identical (low) for the LL and LH conditions. The two conditions differ only in the value of T1 *relative* to the value of T2, which is equal in the LL condition but low in the LH condition. Again, any modulation of LIP activity between these two conditions comprises a signal of relative reward value.

Figures 5A and 5B compare average LIP responses in the LL (blue traces) and LH (green traces) conditions for monkeys A and T, respectively. Note that the blue curves in these figures are the same as the blue curves in Figures 3A and 3B. The data for monkey A show an effect of relative reward similar to that seen in Figure 4A. The green trace drops below the blue trace during the reward cue epoch (black arrow), indicating again that average LIP firing rates fall as the relative value of the target in the RF decreases. The green and blue traces converge again during the motion period and remain together throughout the delay period, indicating a diminished representation of relative reward. As shown in Figure 5B, the effect of relative reward is similar, although weaker, in monkey T (black arrow).

### Quantifying LIP Dynamics: Absolute Value, Relative Value, Motion Coherence and Choice

As is evident from the qualitative evaluation above, LIP population responses are highly dynamic, representing behaviorally relevant variables to differing degrees at different times during the trial. To quantify these trends we applied a multiple-variable, linear regression model to LIP activity over a sliding temporal window as described in Methods. For each LIP neuron we applied the model (equation 3) to the average firing rate over a 50 ms window that was progressively slid, in 1 ms intervals, across the duration of a trial. This generated a time vector of coefficients (β_coh_, β_t1_ and β_t2_ and β_choice_) for each neuron describing the influence of each factor on the mean firing rate at successive time points. Because the values of the different variables were scaled appropriately (−1 to +1), comparison of the coefficients provides an accurate comparison of the effects of each variable on the firing rate of LIP neurons.

**Figure 6:**
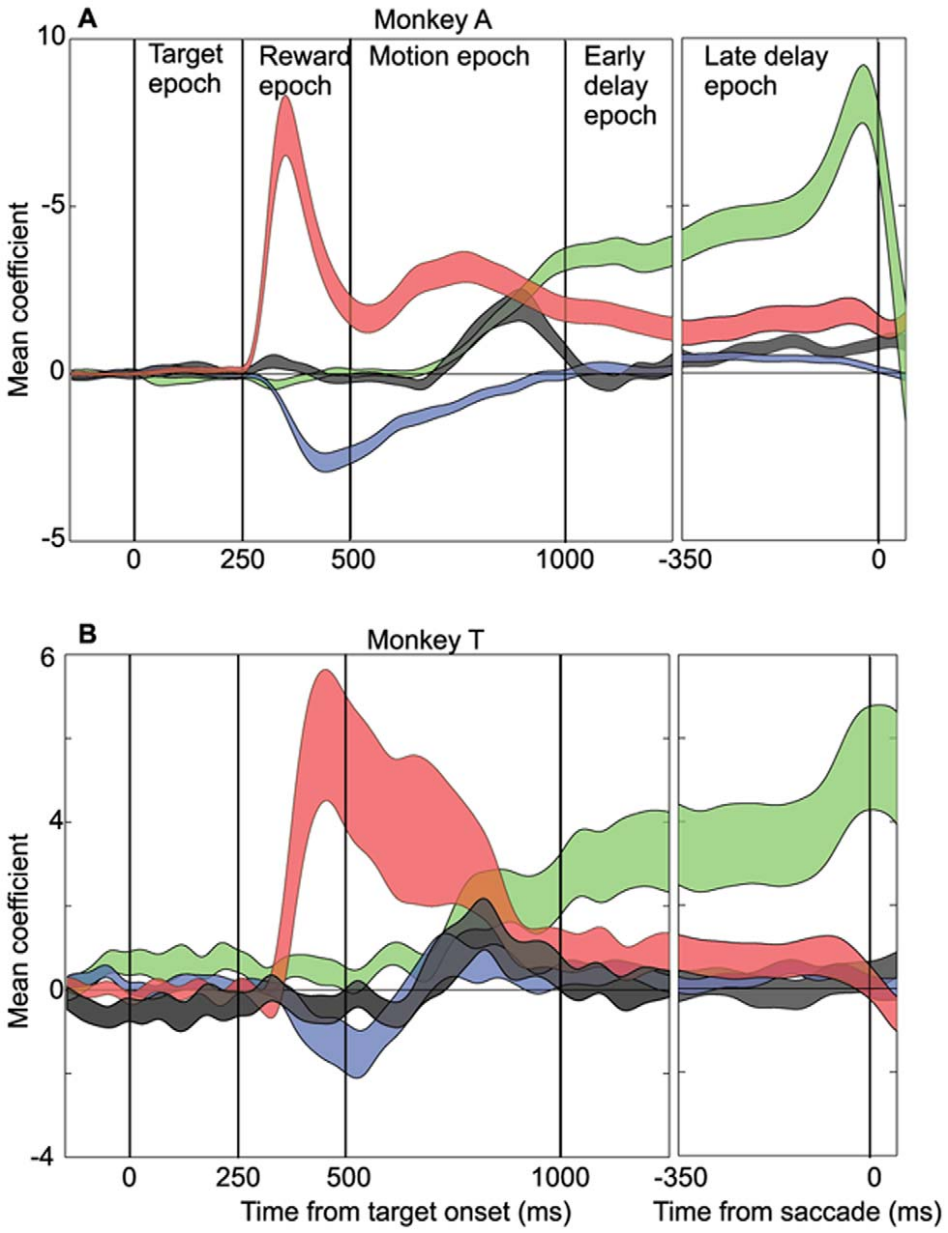
Quantifying the dynamics of absolute value, relative value, motion coherence and choice. **A.** Average regression coefficients from monkey A. **B.** Same data for monkey T. Mean values (±sem) of β_coh_ (black), β_t1_ (red), β_t2_ (blue) and β_choice_ (green) coefficients as a function of time. These coefficients represent the average effect of motion coherence, T1 value, T2 value, and choice on firing rate. They are fit by applying Equation 3 to the average firing rate slid in 1 ms intervals across the duration of the trial. Window width  = 50 ms.

Figures 6A and 6B plot the mean regression coefficients (± s.e.m.) across neurons, for β_coh_ (black), β_t1_ (red), β_t2_ (blue) and β_choice_ (green) as a function of time for monkeys A and T respectively. The same basic trends are evident in the two monkeys, although the coefficients are smaller and more variable in monkey T. The smaller coefficients in monkey T result from the lower overall firing rate modulation (see Figs. 3–5); the greater variance results partly from the smaller sample size (monkey A: n = 51; monkey T: n = 31) and partly from greater intrinsic variability between neurons in this animal.

The quantitative data confirm the general impressions derived from qualitative inspection of the average firing rates in Figures 3–5. The first variables to be reflected in the dynamics of LIP firing rates are the absolute reward value of the target in the RF (β_t1_) and the value of the target outside the RF (β_t2_), which indicates the effect of “relative reward” on firing rate. The effect of T1 value (red curve) rises with very short latency (∼100 msec for monkey T; even faster for monkey A); the effect of T2 value (blue curve) arises more slowly, but is clearly present in both animals by the time of onset of the motion stimulus. The sign of β_t2_ is predominantly negative because a high reward value for T2 decreases the probability of a T1 choice, and thus decreases the firing rate of the LIP neuron. For both animals, a small but significant reversal in the sign of β_t2_ is present later in the trial—during the delay period for monkey A and late in the motion period for monkey T. Notably, both data sets also exhibit a significant but small effect of β_t1_ (absolute reward) throughout the delay period, in contrast to the report of Dorris and Glimcher [32]. This is a significant observation that we will consider further in the Discussion.

Following onset of the motion stimulus, the effects of motion coherence (β_coh_, gray curve) and behavioral choice (β_choice_, green curve) arise—essentially simultaneously given the time resolution of our analysis—with a latency of approximately 200–250 msec, as reported previously [22], [49], [51], [52]. Thus the decision appears to begin forming in the system as soon as evidence about the direction of stimulus motion is present in LIP. Interestingly, the effect of motion coherence abates near the end of the motion period and is completely absent during the delay period. Under the conditions of our experiment, therefore, information about stimulus seems to be discarded once the decision is formed, consistent with previous observations by Roitman and Shadlen ([22]; their Figs. 7B and 7C). As the effects of coherence and target value diminish during the delay period, the effect of choice continues to grow, reaching its peak immediately before the operant saccade. For both monkeys, the peak effects of choice near the end of the trial are nearly equal to the peak effects of absolute value near the beginning.

**Figure 7:**
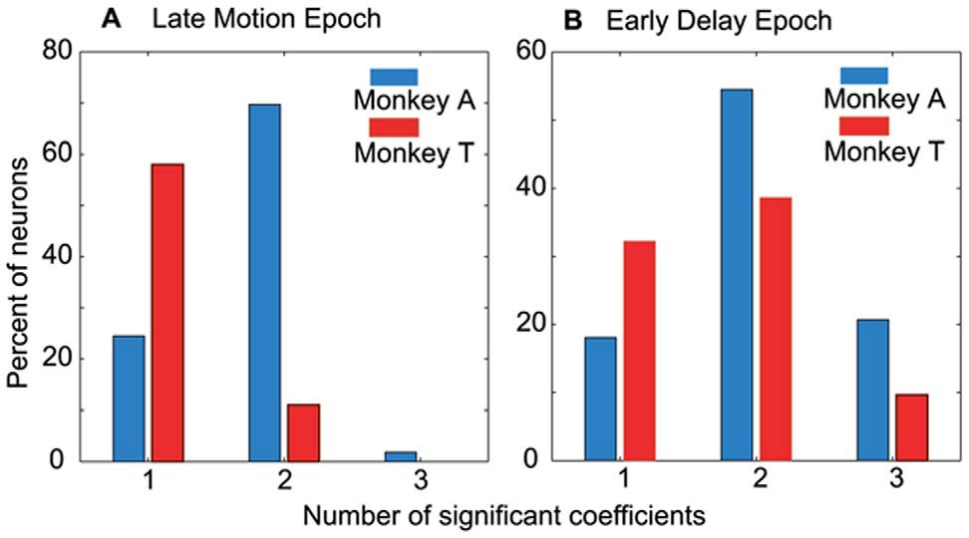
Reward and motion information are multiplexed at the single neuron level. The bars depict the percentage of neurons that are modulated significantly by one, two or three model parameters: T1_val_, T2_val_ or coherence. **A.** Data from the second half of the motion epoch. **B.** Data from the early delay epoch. Red bars: monkey A. Blue bars: monkey B.

Quantitatively, our coherence effects, although highly significant, are smaller than those reported in previous studies of LIP. Shadlen and Newsome [49] reported that a range of coherence from 0% to 51.2% modulated LIP activity by 2.7 spikes/sec for T1 choices and 4.2 spikes/sec for T2 choices. Based on our regression model of LIP activity (Equation 3; β_coh_), we calculate that the range of coherences employed in our study (0%–48%) modulated LIP activity by 2.0 spikes/sec in monkey A and by 0.78 spikes/sec in monkey T. (Because we fit the data with a single model (eq. 3), we did not obtain separate estimates for T1 and T2 choices). Roitman and Shadlen [22] reported substantially larger modulations for the same coherence range: 13.2 and 5.2 spikes per second for T1 and T2 choices, respectively (their Fig. 7B).

### Possible Effects of Eye Movements

The operant saccades to T1 or T2 targets can vary slightly from trial to trial in latency, amplitude, velocity and accuracy. Thus it is possible that these small variations in saccade parameters might account for the change in neural response we have associated with absolute value, relative value, and motion coherence. To assess this possibility, we extended our linear regression model to incorporate various parameters of the operant saccade. For each trial, we calculated five parameters from the stored eye position traces: latency, amplitude, accuracy, maximum speed and duration. We included these factors, along with factors for absolute value, relative value, motion coherence and choice, in an extended regression model given by equation 4 (Methods). We fitted this model, and the original model as well (equation 3), separately to the mean firing rate during three trial epochs: reward cue (250–500 ms), motion (500–1000 ms), and late delay (1000–1550 ms). For all epochs in each monkey, the average values of β_coh_, β_t1_, β_t2_ and β_choice_ were unaffected by inclusion of the saccade parameters in the regression model (paired t-test, p>0.05). Coefficient values sometimes changed significantly for individual experiments after including the saccade parameters in the model, but the direction of the change was not systematic (values could increase as well as decrease) and changes occurred rarely (reward epoch β_t1_: 4.87%, β_t2_: 3.65% of cells; motion epoch β_t1_: 4.87%, β_t2_: 3.65%, β_coh_: 6.09%, β_choice_: 24.29% of cells; delay epoch β_t1_: 7.35%, β_t2_: 10.9%, β_coh_: 1.21%, β_choice_: 8.5% of cells). We therefore conclude that variation in saccade metrics does not explain the response modulation accompanying variations in T1 value, T2 value and motion coherence.

### Do Individual LIP Neurons Integrate Sensory and Value Information

The data in Figure 6 show that LIP neurons, on average, are influenced simultaneously by several variables—absolute value, relative value and motion coherence—and that the relative influence of these variables changes dynamically during the trial. The averaged data presented thus far, however, do not address the issue of whether these variables are similarly mixed at the level of single neurons, or whether population multiplexing emerges from averaging across neurons which are individually more selective. To address this issue, we analyzed data within the late motion (750–1000 ms) and early delay epochs (1000–1300 ms) to determine how many neurons exhibited significant regression coefficients for one factor alone, any two factors, or all three factors. Figure 7A–B depicts the results for the two epochs; data from monkeys A and T are shown in blue and red, respectively. In the late motion epoch a substantial number of neurons in both monkeys were influenced by only one factor, but a roughly equal number of neurons represented multiple factors simultaneously. By the early delay epoch, however, most neurons were influenced by multiple factors. Evidently, the dynamic multiplexing of signals in the average data is characteristic of single LIP neurons as well.

### Relation to the Integrator/Accumulator Model of Decision-Making

For the motion discrimination task with balanced rewards, both psychophysical performance and neural activity in LIP have been modeled by a process in which noisy information is integrated over time [22], [23], [52]. In these models, temporally varying motion information originating in visual area MT is accumulated by competing pools of LIP neurons. In reaction time experiments, a response is triggered when one of the accumulators reaches a bound. In experiments in which the duration of the integration period is fixed by the experimenter, as in the present study, two possibilities have been discussed. According to the first [23], [52], a bound is still used, and the response is determined by the accumulator that reaches the bound first. According to the second (also considered by [11], [12], [15], [23], [42]) the state of the accumulators continues to evolve until the go cue is presented, at which time the accumulator with the largest activation is selected. We couch the following discussion in terms of the first of these two possibilities, returning to the second possibility below.

Applying the bounded integration model to the behavioral paradigm of our experiment, as sketched in Figure 1, two pools of LIP neurons, representing the leftward and rightward saccade targets, would accumulate information from pools of leftward and rightward direction selective neurons in MT. A decision would be reached when the accumulated signal in one pool of LIP neurons reaches the bound.

**Figure 8:**
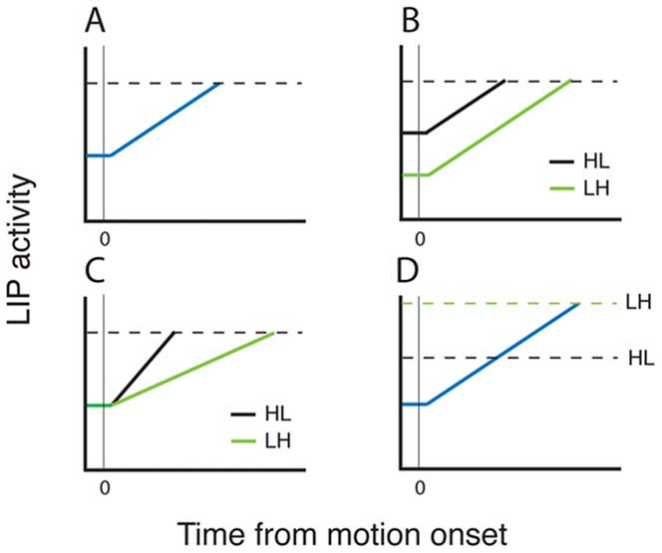
Possible mechanisms underlying the effect of imbalanced payoffs on behavioral choice. **A.** Idealized LIP activity as a function of time during the motion epoch for one spatial location. Time zero indicates the initiation of the motion stimulus. In the model, motion evidence supporting a decision accumulates until it reaches a bound indicated by the dashed lines. **B.** “Two-stage” mechanism. In the first stage, information about payoff size establishes the initial offset of the accumulator, which, in the imbalanced payoff conditions (HL and LH), is biased in favor of the spatial location of the high payoff target. In the second stage, motion information accumulates to a fixed bound, as in A. **C.** “Drift rate” mechanism. The accumulator offset is identical for all payoff conditions, but payoff information is incorporated into the drift rate of the accumulation process, again biasing the process in favor of the high payoff target. **D.** Payoff information affects neither the offset nor the drift rate, but rather exerts its effect through adjustment of the decision bound. HL  =  high-low reward condition (large payoff target in the LIP response field; small payoff target in the opposite hemifield). LH  =  low-high reward condition (small payoff target in the LIP response field).

The accumulation process is schematized by the cartoon of Figure 8A. This trace illustrates an idealized average firing rate for one pool of LIP neurons under balanced reward conditions. LIP activity departs from steady state shortly following the onset of the motion stimulus (time 0), integrating incoming motion information until a bound (dashed line) is reached. Under balanced reward conditions, the two accumulators compete on equal footing (the other accumulator is not shown), and the outcome of the decision process is therefore determined by the relative strength of the motion input to the two LIP accumulators plus stochastic variability in the sensory evidence and in the accumulation process itself. In the unbalanced reward conditions (HL and LH), decisions are biased strongly toward the higher value target (Fig. 2), but the neural mechanisms underlying this behavioral bias are unknown.

Figures 8B–D illustrate three possible mechanisms suggested by Diederich and Bussmeyer [1] that could account for the choice bias in the unbalanced reward conditions. The first possibility (Fig. 8B) is that the reward cue produces an offset in the initial value of the accumulator, granting a relative advantage to the accumulator corresponding to the high value target. In the HL condition, for example, the high value target is in the RF of the LIP pool under study, and the offset is thus positive relative to the other accumulator, which is in the LH condition (compare the black and green traces). The accumulator with the positive offset will therefore tend to reach the bound sooner, resulting in more choices of the RF target in the HL condition. Conversely, the RF target will be at a relative disadvantage in the LH condition (green trace), resulting in more choices of the non-RF target. A second possibility (Fig. 8C) is that the reward information has no effect on the starting point of the accumulation process, but rather affects the drift rate of the diffusion process by contributing an additional input to the accumulator when the high value target is in the RF (black trace) and/or a negative input to the accumulator when the low value target is in the RF (green trace). An effect of payoff information on drift rate would increase the slope of the accumulator activation curve for the HL condition and/or decrease the slope for the LH condition. Both effects would increase the likelihood of a choice of the high value target. A third possibility (Fig. 8D) is that the reward information affects the bound, not the state of the accumulators. Thus the bound would be lowered when the high value target is positioned in the RF (HL bound, Fig. 8D) and raised when the low value target is in the RF. These potential mechanisms, of course, are not mutually exclusive, nor are they exhaustive.

**Figure 9:**
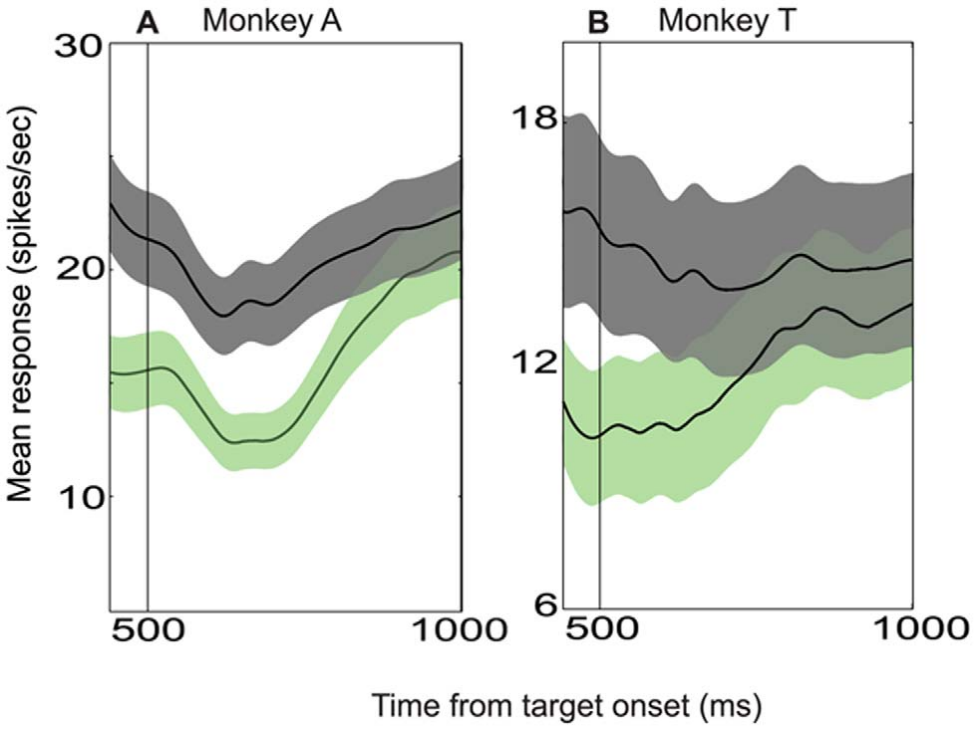
Unbalanced rewards results in an offset to the starting point of the accumulation process. **A.** Average LIP firing rate (±sem) as a function of time for the HL (black) and LH (green) reward conditions (monkey A, T1 choices only). Activity is averaged across all coherences. The black and green curves are replotted from Figs. 4A and 5A (respectively), expanding the horizontal scale to emphasize the interval at and following the onset of stimulus motion (time 500). **B.** Equivalent data for monkey T. Traces are replotted from Figs. 4B and 5B.

Our LIP data allow us to evaluate contrasting predictions of the first two candidate mechanisms. Figure 9 illustrates data from monkey A and monkey T that are directly analogous to the idealized traces for the HL and LH conditions in Figure 8B–C. These data are for T1 choices and appeared previously in Figures 4 and 5; the key elements of the data are reproduced here for ease of comparison, focusing on the motion presentation epoch (time 500–1000) when the accumulation process actually occurs. For both monkeys, it is clear that the traces are offset with the expected sign during the first 200 milliseconds of the motion epoch, confirming the prediction of the “offset” mechanism illustrated in Figure 8B.

The data in Figure 9 do not conform to the prediction of the “drift rate” mechanism in Figure 8C. In fact, the slope appears *shallower* for the HL condition compared to the LH condition. However, these traces are averaged across all motion coherences for each animal (T1 choices only). The HL traces are thus enriched in low coherence stimuli compared to the LH traces because of the strong behavioral bias toward T1 choices in the HL condition. In the LH condition, there are fewer T1 choices overall, and these T1 choices tend to occur when the motion information is sufficiently strong (high positive coherences) to override the reward bias. Strong positive coherences will drive the accumulation process more rapidly than weaker coherences, leading to the slope effect observed in Figure 9.

**Figure 10:**
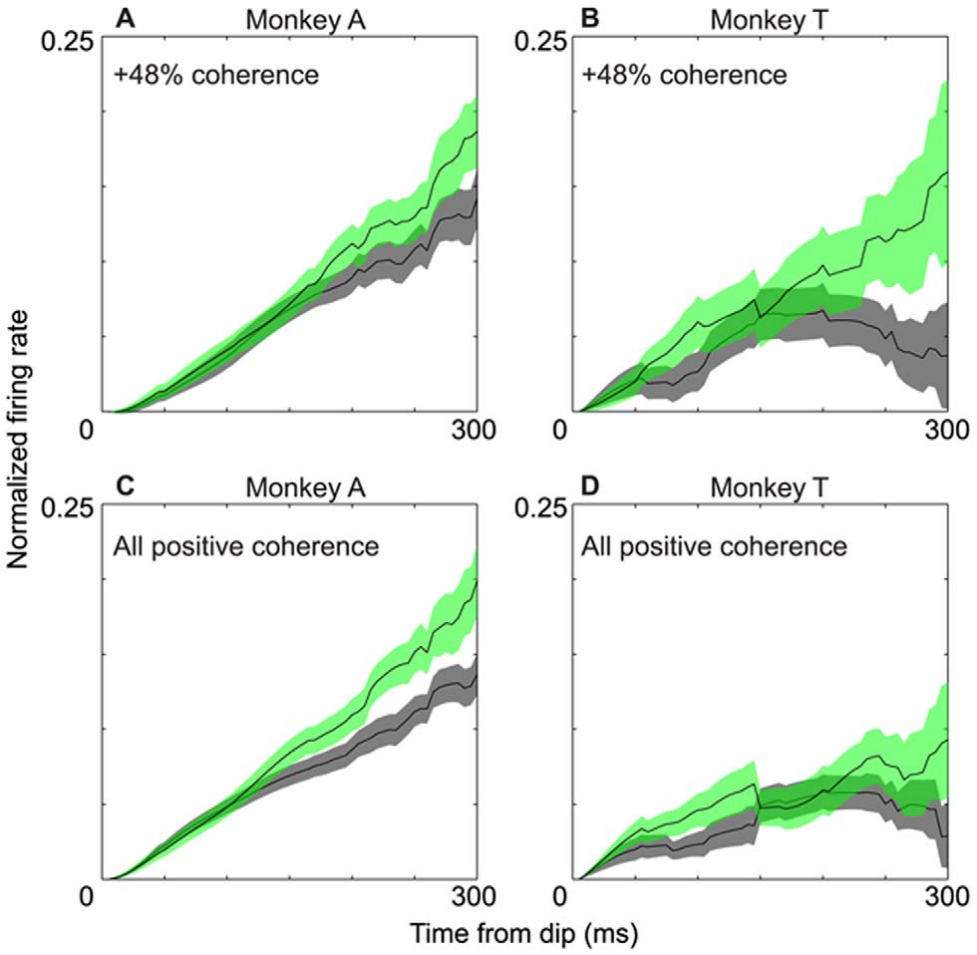
Rate of accumulation for the unbalanced reward conditions. **A–B.** Normalized firing rates (±sem) for a single motion condition (+48% coherence), averaged across the population of neurons from monkey A and monkey T, respectively. All data are from trials ending in a T1 choice. **C–D.** As in A–B, but averaged across all positive coherences. The HL condition is depicted in black, the LH condition in green. Time zero is the time of the initial “dip” in firing rate following onset of the motion signal, identified separately for each neuron (see Methods).

To factor out the effect of coherence on the accumulation slopes, we first normalized firing rates within each stimulus condition (signed coherence) before averaging across trials to obtain PSTH's for each reward condition (see Methods for details). To visualize the normalized data, we then averaged the resulting PSTH's for a given coherence across the population of neurons for the HL and LH reward conditions. Figures 10A and 10B show the results of this analysis for +48% coherence, which resulted in T1 choices on nearly every trial for both monkeys (Figs. 2C and 2D). On the horizontal axis, the trials are aligned to the time of the ‘dip’ measured for each neuron (see Methods). On the vertical axis, all firing rates begin at zero at the dip, as described in Methods. Thus the curves illustrate the accumulation process for the HL (black) and LH (green) reward conditions from the time of the dip to the end of the measurement window (defined in Methods). The basic result is clear and somewhat surprising, even though the data are noisier for monkey T (due in part to the smaller number of neurons contributing to the analysis). The traces for the two reward conditions are indistinguishable for the first 200 milliseconds following the dip, contradicting the prediction of the drift rate mechanism in Figure 8C. Similar trends are evident when the data are averaged across all positive coherences as illustrated in Figures 10C and 10D. The slope for HL still becomes shallower than the slope for LH, but only toward the end of the motion integration period. To confirm this impression statistically, we measured the slope of the LIP PSTH's in the HL and LH conditions for each neuron, after averaging traces like those in Figures 10A and 10B across all positive coherences for each cell (see Methods). During the first 200 milliseconds following the dip, there was no significant difference in the distribution of slopes in the HL and LH conditions for either monkey (paired t-test, p>0.05 for both monkeys). When slopes were calculated across the entire motion integration period (as defined in Methods), however, the distributions differed significantly between the HL and LH conditions in both monkeys, with slopes being *shallower* in the HL condition (paired t-test, p<0.002 for monkey A, p<0.02 for monkey T).

The evidence in Figures 9 and 10 provides direct support for the view that the hypothesized LIP accumulator starts higher in the HL condition than in the LH condition, and that the drift rate of the accumulators is initially unaffected by the reward condition. As we shall discuss more fully below, the shallower slope for the HL condition toward the end of the integration period is consistent with the presence of an integration bound, which is reached sooner in the HL than in the LH condition. With this encouragement, we conducted mathematical analysis and simulations that we now describe to determine whether a bounded integration model can account for our experimental data—both behavioral and physiological.

### Mathematical Analysis and Simulation

For several reasons, the analysis above points toward a bounded integration model, with relative reward affecting the starting point of the integration process. However, the behavioral data pose an immediate challenge to the bounded integration model. The presence of the bound renders an integration process imperfect because it limits the amount of information accumulated, and this can produce distortion in the pattern of behavioral results. This distortion is most obvious if we consider the HH vs. LL conditions in the data for monkey A. These data indicate that the accumulators start closer to the bound in HH than they do in the LL condition. In that case, the bound is reached sooner on average in the HH condition; less information is therefore integrated, with the result that behavioral performance should be less accurate. Yet there is no difference in the behavioral performance between the HH and the LL conditions (Fig. 2).

To address this issue, we must consider two distinct possible sources of variability in the decision process. The first of these—and the one generally receiving the greater emphasis in the literature—is moment-by-moment noise in the input to the accumulators. Let us consider an accumulator model with two accumulators racing to a decision bound. One can characterize the input to each accumulator by the following simple equation:

Equation 5:
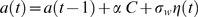
where *a(t)* represents the activation of the accumulator at time t, α is an integration rate parameter (it also indicates the sensitivity to stimulus), C represents the coherence such that positive values excite the accumulator, η(t) represents a sample of noise from the standard normal distribution taken at time t, and σ_w_ is a scale factor representing the standard deviation of the within-trial, moment-by-moment noise in the integration process. For our case, we are considering a situation in which there are two accumulators, one for each alternative. Equation 5 applies to the accumulator corresponding to the neuron recorded in the physiological experiment. For the other accumulator, C is replaced by –C, so that values exciting one accumulator are inhibiting the other. For such an accumulator model, the distortions discussed above arise and preliminary simulations (not shown) resulted in very poor fits to the behavioral data.

While some models include only the moment-by-moment variability discussed above, others include a second source of variability, namely between-trial variability in the strength of the sensory evidence reaching the accumulators. This idea was first employed by Ratcliff [5] in accounting for human behavioral data, and has since been incorporated in many other models, including the LATER model, which has been used to account for correlation between the slope of activation in FEF and latency of eye movement responses [18], [53] [54]. In our case, we capture between-trial variability in the motion-dependent input signal to the accumulators by assuming that the value of C is perturbed, for the duration of a whole trial, by a sample from the standard normal distribution scaled by σ_b_ (the between-trial standard deviation parameter), so that the integration equation becomes:

Equation 6:
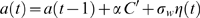
where *C'  =  C + σ_b_η*, represents the perturbed value of *C*. For the other accumulator we replace *C'* with *–C'*, so that the same perturbation affects the input to both accumulators. Importantly, we do not ascribe this between-trial variability to any particular cortical area or processing stage. It may originate in the motion output of MT (due, for example, to the stochastic stimuli employed in these experiments) or to any additional stage of the pathways linking MT to LIP.

A consequence of between-trial variability is that the accuracy of the outcome of the information integration process is less dependent on the duration of integration. It can be shown that the mean of the accumulated sensory information is a simple linear function of *t*,

Equation 7:
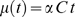
while the standard deviation in the accumulated information after *t* seconds is given by

Equation 8:
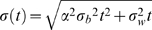
As a result the signal-to-noise ratio can be expressed:

Equation 9:
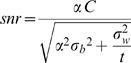
Two points follow from this equation. First, if between-trial variability is high relative to within-trial variability, the signal-to-noise ratio can easily be dominated by the between-trial variability. Second, as time goes by, the relative importance of within-trial variance decreases. Thus, if between-trial variability is relatively high, as long as the accumulation process starts far enough from the decision bound, starting even further from the decision bound can make very little difference in the accuracy of behavioral choices. Based on this insight, our bounded integration model incorporates the assumption that between-trial variability is relatively high.

### Simulation Model Details

We simulate data from monkey A, for whom we have the largest and cleanest data set. As we shall see, it is possible to provide a good qualitative fit to the data from this monkey within the framework of the ideas described above. After considering monkey A, we will return to consider the data from monkey T, which is both noisier and more perplexing in certain ways.

**Figure 11:**
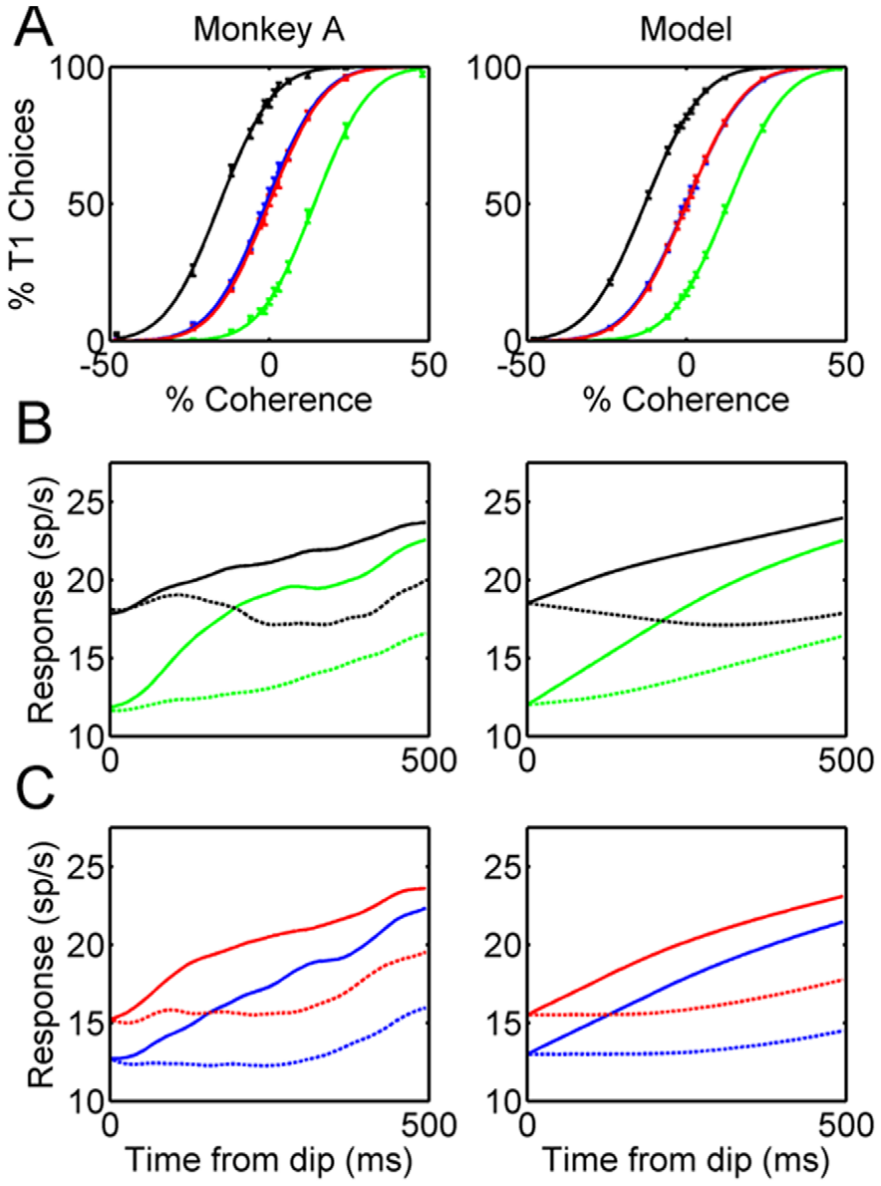
A model account of the neural and behavioral observations in Monkey A. **A.** Empirically observed behavioral data from monkey A (left) and simulated behavioral results (right) of the competing accumulator model described in the text. Four colors indicate the four reward conditions: HH (red), LL (blue), HL (black; LH (green). **B.** Empirically observed physiological data from monkey A (left) and simulated physiological results (right) for the imbalanced reward conditions. Solid lines indicated trials ending in T1 choices; dashed lines illustrate trials ending in T2 choices. Color code is the same as in the top panels. **C.** Empirically observed physiological data from monkey A (left) and simulated physiological results (right) for the balanced reward conditions. Solid and dashed lines, and the color code, are the same as in the preceding panels. Parameter values used in the reported fits are as follows: *b* = 10; *a* = 0.5; *σ_b_* = 14; *σ_w_* = 1, θ = 22. Values are in units of seconds and Hertz.

Our model shares many features with the LIP portion of the model presented by Mazurek et al [23], but we do not directly simulate the sensory inputs from MT. Rather, we simply consider the input to the accumulators to have both within and between trial variability as indicated above. Our simulation incorporates the following features:

1. The starting point of each of the two accumulators is affected by both relative and absolute reward. In our simulations, the starting point of the T1 accumulator is initialized to the empirically observed activation level at the time the motion stimulus begins to affect activation (the “dip”), approximately 200 msec after motion onset. The starting value assigned to the T2 accumulator is based on the empirically measured T1 values, assuming that the values for T2 are symmetric to those measured for T1: (T2 HL  =  T1 LH, T2 LH  =  T1 HL; T2 LL  =  T1 LL, T2 HH  =  T1 HH).
2. The information accumulation process is affected by both within- and between-trial variability and also by an urgency signal. The activation of the T1 accumulator is updated according to:
  Equation 10:
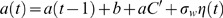
Where, as previously discussed, *C'* is equal to the stimulus coherence C perturbed by a sample of Gaussian noise with standard deviation *σ_b_*. For the T2 accumulator, *C'* is replaced with –*C'*. Within-trial, moment-to-moment Gaussian noise with standard deviation *σ_w_* is added independently to each of the two accumulators. The parameter *b* is a positive constant reflecting an overall tendency for activation to increase during the motion period, corresponding to the “urgency” signal of Mazurek, et al. [23] and other investigators. (See the caption of Figure 11 for parameter values.)
3. Information integration occurs for a period of time equal to the duration of the motion stimulus unless the bound is reached before the end of the integration period (see next). In comparing the simulation to data, we treat integration as beginning after a 200 msec propagation delay, so that the simulated processing interval corresponds to the period from 200 to 700 msec post stimulus onset approximately.
4. Integration is bounded, so that when the activation of one accumulator reaches the bound value θ, integration of sensory information in both accumulators ceases. The bound is viewed, not as an upper limit on neural activity, but as an internal benchmark on activation, such that when this benchmark is reached, the process of integration ceases, affecting both accumulators equally. Although integration ceases when the bound is reached, the influence of the urgency signal continues until the end of the trial.
5. The behavioral choice is assigned to the accumulator whose activation value is highest at the end of the motion period. In cases where the bound is reached, the winning accumulator always corresponds to the accumulator that reached the bound and caused integration to cease.
6. Parameter values were estimated according to the following procedure. Parameters *a* and *σ_b_* were first approximated by selecting values that permitted a good fit to the behavioral data ignoring the effect of the bound and of the within-trial variability *σ_w_*, which has a negligible effect after 500 msec of integration. These approximate values could be directly estimated without the need to run the simulation. Estimation of the urgency signal, *b*, and the activation bound value, θ, required searching the parameter space via simulation. Simulation results reported in the figure were based on 25000 simulated trials for each of the 52 combinations of stimulus and reward conditions.

### Simulation Results

Figure 11 shows the results of the simulation (right column), along with the comparable psychophysical and neural data from Monkey A (left column). The model captures several important features of both the behavioral and neural data. We begin with a consideration of the behavioral performance: The probability of a T1 choice as a function of coherence is identical in the HH and LL conditions (Fig. 11A), even though the accumulation process starts at a higher level in the HH than in the LL condition (Fig. 11C); the choice curves for HL and LH conditions are simply shifted to the left or right compared to either the HH and LL curves, in the simulation as in the behavioral data. These patterns are expected based on our analysis above. The high between-trial variability in the drift parameter is the dominant source of variability affecting the choice outcome, so that the outcome is relatively immune to the location of the bound. In essence, the slopes of the behavioral curves depend on the ratio of the parameters *α* and σ_b_, and the bound on integration has relatively little importance.

The relative positions of the curves along the x-axis reflect the difference in the starting values of the accumulators, which persists throughout the integration period. Ignoring the bound, and taking the choice to be determined by the accumulator that is more active at the end of the motion period, the magnitude of the shift can be directly calculated from the ratio *S_d_/σ*(500), where *S_d_* is the difference in the starting points of the accumulators (*S_d_ = S1–S2*) and σ(500) is the standard deviation of the difference in activation of the two accumulators at the end of the motion period, see Equation 8. Once again, because of the high between-trial variability, the presence of the bound and the within-trial variability has a negligible effect on behavior, with the result that the curves are simply shifted left or right by an amount determined by the above ratio.

Because the bound does not impact behavior, an alternative account of the behavioral data would be to suppose that there is no bound on the integration process, and that the monkey simply chooses the most active accumulator at the time he receives the signal to respond. We would not rule out such an account, and we consider such a possibility further in the Discussion. However, including a bound on integration helps us to account for many features of the physiological data, which we now consider:

1. For trials ending in T1 choices, the model captures the negative acceleration (i.e. saturation) of the slopes of the neural activation curves near the end of the motion integration period (Fig. 11 B and C). This negative acceleration reflects the effects of reaching the decision bound, which occurs on many but not all trials. For the activation bound parameter value that was used in the simulation, one of the two accumulators reaches a bound on approximately 70% of the trials on average, although the fraction increases with the absolute value of the stimulus coherence C and when the direction of motion is congruent with reward bias. The bound is reached at different times on different trials, accounting for the gradual flattening of all four activation curves for T1 choices.
2. In the model as in the data, the neural activation curves for T1 choices in the HL and LH conditions converge noticeably but not completely during the motion integration period (Fig. 11B, compare the solid green and solid black curves). The difference in the T1 activation curves is due in part to the different mixture of coherences contributing to T1 choice trials as discussed above in conjunction with Figure 10, and also to the fact that activations tend to reach the T1 bound, and thus stop growing, sooner (and more often) on average for HL than for LH choices. Convergence to exactly the same level would be expected if the bound was reached on all T1 choice trials. In the model, however, the bound is not actually reached on all trials (point 4 above); on these trials the decision is simply cast in favor of the accumulator with the highest activation level (point 5 above). Thus the T1 activation curves in the model tend toward convergence in the HL and LH conditions without actually reaching the same level.
3. In both the model and the data, the HL and LH activation curves converge for T2 choices as well (Fig. 11B). The same factors that affect convergence of the T1 choice curves are also in play in the T2 choice curves.
4. In the model, there is a subtle trend toward convergence of the HH and LL curves for T1 choices (Fig. 11C); this effect is due to the fact that accumulation terminates at the bound for the T1 accumulator sooner on average (and on a higher number of trials) in the HH condition. The effect is subtle because the initial offset between the HH and LL curves is smaller than between the HL and LH curves, resulting in a smaller difference in termination times, and because there is no difference in the mix of coherence values terminating in T1 choice for the HH and LL conditions. In the data, the HH and LL curves are similar in shape for T1 choices, as in the simulation; the difference between the curves seems slightly smaller toward the end of integration than at the beginning. While the effect in the data is unlikely to be statistically reliable, the subtlety of the effect in the simulation is such that a statistically reliable effect in the data would not be expected.
5. The model reproduces the rising slope of the accumulation curves for T2 choices near the end of the motion period in all four reward conditions (Fig. 11B and C, dotted curves). This is due to the “urgency” signal represented by parameter *b* in Equation 10, which continues to affect activation in the model after integration stops. The urgency signal captures the intuition that a premium exists on reaching decisions within a finite time, even on low coherence trials when evidence may accumulate very slowly [23]. Thus both accumulators are driven toward their bounds at a slow but steady rate throughout the trial, independent of evidence accumulation. This factor is less apparent earlier in the trial, where activations reflect both the stimulus effect and the urgency signal.

Overall, the simulation captures both the behavioral data and most of the main features of the physiological data from monkey A. Thus, for this monkey at least, the behavioral and physiological findings appear to be consistent with the hypothesis that reward affects the starting point of an integration process that is subject to high between-trial variability and that employs a decision bound placed such that it is reached only on a subset of trials.

We now consider briefly whether the model described here can account for the data from monkey T. As indicated earlier, several features of monkey T's data are consistent with monkey A's data and thus with the model: 1) both absolute and relative reward effects are present (Figs. 4–6), 2) the offset in the starting point of the accumulation process is clear (Fig. 9B), and 3) the dynamics of the accumulation process are similar to monkey A once the effects of coherence are controlled for (Fig. 10). The most perplexing aspect of monkey T's data is seen most clearly in Figure 4. The firing rate trace for the HH condition (solid red curve) is initially lower than the trace for the HL condition (solid black curve), consistent with the relative reward effect. About 200 msec into the motion viewing period, however, the traces reverse order, with average firing rate becoming higher for the HH condition, and the reversal holds for the duration of the trial. This crossover would not be expected in our model; as in the data and the simulation of monkey A, we would expect the curve from the HH condition to converge toward, but not cross, the curve for the HL condition. The cross-over is anomalous, not only from the point of view of the bounded integration model, but also from the point of view of the overall pattern of findings, in which high relative reward of the RF target is typically associated with greater LIP activity.

A variety of approaches might be taken to account for this perplexing result from monkey T. For example, the data could be explained if we relax the assumption that the integration bound is kept the same across all of the reward conditions. If the bound were adjusted upward in the HH condition, keeping it low (and approximately constant) in all of the other reward conditions, the model might then provide a reasonably good approximate account of all facets of monkey T's data. We emphasize that this is only one possible account, and we do not specifically wish to advocate for it. We mention it only to make the point that there is at least one way to explain the anomalous results seen in Figure 4 with a bounded integration framework.

## Discussion

We examined the dynamics of neural activity in LIP while rhesus monkeys performed a 2AFC motion discrimination task under conditions of equal and unequal payoffs signaled at the beginning of the trial. As reported previously [42], psychophysical performance was indistinguishable during the two balanced reward conditions (HH and LL), but imbalanced rewards (HL and LH) biased the monkeys' choices strongly toward the target associated with the higher reward. Thus the *absolute* value of the target in the RF did not affect choices in our paradigm, whereas the *relative* value of the RF target affected choices substantially. The coherence of the visual stimulus also exerted a strong effect on choices, as evidenced by the well-formed psychometric functions in Figure 2.

In the Introduction we posed four questions about neural processing in LIP to be addressed in these experiments, two descriptive and two mechanistic. At the descriptive level, we find that single LIP neurons reflect all three variables that we manipulated—absolute value, relative value and coherence—in addition to the well-known representation of behavioral choice (e.g. [51]). The neural representation of these variables was dynamic, with each variable influencing average firing rate in a stereotyped sequence at the population level (Fig. 6). Upon presentation of the reward cue, LIP neurons responded rapidly to the absolute value of the target in the response field, with higher value targets generating higher firing rates. Within 200 ms, this neural representation of absolute value was modulated by the value of the target outside the response field, creating a representation of the relative value of the RF target. Importantly, the representation of relative value was most pronounced at the start of the motion epoch, consistent with a mechanistic effect of biasing the starting point of the motion integration process (see below). As the motion epoch developed, however, the representation of both relative and absolute value faded and the effects of motion coherence and behavioral choice emerged. The representation of choice quickly dominated LIP responses and persisted through the time of the saccade. Interestingly, the representation of absolute value persisted in reduced form until the end of the trial, even though it exerted no effect on the psychometric functions. The signals we observed in LIP were typically multiplexed at the single neuron level; a large proportion of neurons in both animals were influenced by multiple variables, especially in the early delay period (Fig. 7).

Most of these descriptive observations have precedents in the existing literature. Modulation of LIP visual responses by absolute reward level has been reported previously [30], [40], [41], as have the effects of relative reward [30], [32], [34], motion coherence [22], [49], [51], [52], [55] and behavioral choice [30], [51]. A single point of conflict with the previous literature is our observation that a dramatic effect of absolute reward is evident at the onset of the cue period and persists at a reduced level until the end of the trial. This effect is evident in the comparison of HH and LL conditions in the average PSTH's of Figure 3 (for both T1 and T2 choices) as well as in the regression results of Figure 6. In contrast, Dorris and Glimcher [32], using a two-alternative, forced-choice “inspection game”, reported that LIP activity represents relative value only, with no difference in firing rate observed between blocks of trials with standard vs. double reward size (their Fig. 10). Dorris and Glimcher thus concluded that LIP encodes the “relative subjective desirability” of different spatial locations.

It seems likely that the effect of absolute reward is more salient in our data because our behavioral paradigm required an explicit evaluation of payoff size on every trial due to the random interleaving of reward conditions. In the behavioral paradigm used by Dorris and Glimcher, overall reward size was kept constant throughout an entire block of trials; the variable that the monkey needed to judge from trial to trial was the likelihood of being “inspected” by the computer opponent, with the consequence of receiving no reward at all. In contrast, successful harvesting in our paradigm depended strongly on accurate evaluation of the constantly changing payoffs associated with each possible choice. The presence of the absolute reward effect in our data indicates that—in the context of our behavioral paradigm—LIP activity at any one spatial location does not, by itself, completely specify the value of that location relative to other locations. Nor does it completely specify the probability of choosing a particular option on a given trial (e.g. [34], [56]). More generally, however, we agree with Dorris and Glimcher and with Sugrue et al. that relative value is easily calculated from neural activity in LIP; it simply requires a comparison of activity at the two locations in LIP representing the choice targets (see below).

### Mechanism of the Effect of Payoffs on Behavioral Choice

The most important insight provided by our study concerns the mechanistic basis of the effect of unequal payoffs on behavioral choice. In a psychophysical and modeling study of the effect of payoffs on discrimination near sensory threshold, Diederich and Busemeyer [1] examined the predictions of three possible mechanisms. Their “two-stage processing hypothesis” corresponds approximately to the cartoon in Figure 8B. This hypothesis postulates a first-stage accumulator that accrues information about the relative payoffs of the two choices, which results in an offset to the starting points of the two sensory accumulators (second stage). Their “drift-rate change hypothesis” corresponds to the cartoon in Figure 8C, in which payoff information exerts its effects via the rate of accumulation of sensory information in the diffusion process, and their “bound-change hypothesis” corresponds to the cartoon in Figure 8D. Diederich and Busemeyer found that the two-stage processing hypothesis accounted best for their psychophysical data, suggesting that payoff information influences choices through an offset in the starting point of the sensory accumulation process. This conclusion is consistent with the microstimulation data of Hanks and colleagues [27], which suggests that artificial activation of LIP during the motion discrimination task influences the initial offset of the neural accumulators.

Our electrophysiological data strongly support the offset mechanism proposed by Diederich and Busemeyer and by Hanks and colleagues, directly demonstrating the hypothesized offset at the initial point of accumulation of sensory evidence (the “dip”—Fig. 9; Fig. 11B and C). In addition, our data argue strongly against the drift-rate change hypothesis, showing that the initial rates of accumulation are nearly identical for the HL and LH conditions, and that the eventual direction of divergence (LH slope steeper than HL) is opposite to the prediction of the drift-rate change hypothesis (Fig. 10).

Because we used a fixed-interval psychophysical procedure, not a reaction time procedure, we are unable to evaluate directly the predictions of the bound-change hypothesis. However, our simulations showed that a bound-change is not necessary to account for our data, at least for the case of monkey A, the monkey from who we have the largest and cleanest data set. The primary features of this data set, both behavioral and neural, can be reproduced by a simple model that incorporates 1) a payoff-driven offset in the starting point of accumulation, 2) intra- and inter-trial noise in the input to the accumulators, and 3) the assumption that accumulation ceases and a decision is made when either accumulator reaches a stationary bound. It should be noted that there may be other ways to account for the data from monkey A that omit a bound from the model. In our fixed-interval task, for example, accumulation could continue until the end of the stimulus period, and the decision could be reached from a simple comparison of the values of the two accumulators. Although further simulations would be required to verify this, it is likely that, with additional assumptions, such an approach could account for many aspects of data.

Our simulations make sense of several otherwise puzzling aspects of our behavioral and neural data. First, the simulations defuse any concern about the presence of an absolute reward signal during the critical motion and early delay periods. At first blush, the absolute reward signal seems worrisome because behavioral decisions appear to be based solely on the relative value of the two targets (Fig. 2). The presence of an irrelevant absolute reward signal during the critical time of decision formation might lead one to doubt whether LIP neurons actually contribute to the primary process of decision formation. Our simulations of the results from monkey A, however, nicely reproduce both the behavioral data (Fig. 11A) and the absolute reward effect (Fig. 11C), demonstrating that the two data sets are compatible with the notion of bounded accumulation in LIP. In the model, the absolute reward effect exerts little effect on simulated behavioral choices because the absolute value signal is present in *both* accumulators (T1 and T2) during the accumulation process and thus grants no advantage to either accumulator. In essence, the relative value that governs decisions results from the relative activation of the two accumulators; any signal that is common to both will have little or no effect on behavioral choices.

Second, the simulations rationalize the results of the otherwise puzzling slope analysis of the T1 accumulator under the unequal reward conditions (Fig. 10). Not only are these accumulation slopes inconsistent with the drift-rate change hypothesis, they actually trend in the opposite direction, with slopes rising more steeply for the LH than for the HL conditions. The simulation shows, however, that this is a logical result of the model assumption that all accumulation ceases when the bound is reached by either accumulator. Because the bound is reached earlier on average and more often in the HL than the LH conditions (for T1 choices), the HL curve saturates more strongly than the LH curve, producing the tendency toward convergence.

Finally, the model accounts well for the behavioral observation that performance is nearly identical under the HH and LL reward conditions. One might have expected performance to be worse in the HH condition because of the closer proximity of the initial value of the accumulator to the sound, which should permit within-trial noise in the accumulation process to exert a greater effect on the final decisions. In our simulations, however, this effect is neutralized by the presence of between-trial noise in the motion-dependent input signal to the accumulators, which renders behavioral accuracy relatively insensitive to the distance between the starting point and the bound. Between-trial noise in the reliability of the motion-dependent input signal may arise in part from the different patterns of dots employed on each trial for a given motion coherence. The value of our between-trial variability parameter is fairly large (twice as large, for example, as the fitted value in Ditterich, 2006). Holding all other features of our model constant, the value of this parameter is rather tightly constrained by the data. It is difficult to know whether this discrepancy should be a concern, given that our experiment differed in many details from the one modeled by Ditterich, and given that parameter values can differ substantially between individual monkeys. A smaller value for between-trial variability might fit our data if other features of the model were adjusted. Whether such adjustments would lead to a better account over all should be explored in further research.

As noted at the end of the results section, the clear picture presented in the data from monkey A is not quite so clear in monkey T. However, the data from monkey T exhibit most of the same features observed in monkey A. Most importantly, both absolute and relative reward effects are present in LIP activity at the onset of the motion period, consistent with the idea that reward shifts the starting place of an information integration process. The cross-over of neural activity in the comparison of the HL and HH conditions is unexpected, but can be accounted for within the bounded integration theory if the bound were set higher in the HH condition than in the other reward conditions.

### Interpretations of LIP Activity

Substantial energy, both conceptual and experimental, has been expended in seeking a general theory of LIP computation [57], [58]. Early efforts attempted to distinguish between interpretations that were rooted in sensory (attention) versus motor (intention) perspectives [59], [60], [61], [62]. Although this controversy inspired a series of heroic experiments attempting to distinguish between these perspectives, the effort was ultimately inconclusive, in part because researchers could not agree on operational definitions of the postulated cognitive processes. At a deeper level, however, the attempt to distinguish between visuospatial attention and intention to move the eyes was probably doomed from the start because the neural systems that mediate attention and intention are fundamentally inseparable. Evidence emerging in the last decade suggests strongly that the neural structures that mediate orienting movements of the eyes and head also mediate covert spatial attention [63], [64], [65], [66], [67], [68]. It now seems likely that covert spatial attention (attending to a spatial location different from the current direction of gaze) is an advanced adaptation of basic oculomotor circuitry, and is thus unlikely to be cleanly separable from internal preparation to move the eyes, no matter how heroic the experimental design.

A similar problem may exist with more recent suggestions [41], [69], [70] that reward and value effects that have been documented in recent studies of LIP [30], [32], [34] should be subsumed under the rubric of attention. Maunsell [69], for example, has pointed out that studies in experimental animals deliberately manipulate attention by enforcing reward contingencies, and that studies of reward value rarely try to exclude the possibility that the observed neural effects are actually the result of attention covarying monotonically with reward value. It is thus possible that “some studies of attention and reward might have been looking at exactly the same neural signals” [69]. For example, the biasing effects of unequal rewards demonstrated in the current study could be easily interpreted as effects of attention. Maunsell suggests experiments that might disentangle attention and reward value, and some recent studies have attempted to do just that [41], [70]. We are open to the possibility of a clean dissociation, but we are wary of the field again becoming enmeshed in a controversy that is fundamentally irresolvable. This danger is particularly acute if the notion of “value” is extended (as it probably should be) beyond the promise of immediate rewards to include acquisition of information pertinent to acquiring rewards in the future (e.g. [71]). From an evolutionary point of view, many of the phenomena that we refer to as attention may have arisen from circuitry that originally implemented direct reward-seeking behaviors, and these neural circuits may still overlap considerably within our own brains, rendering clean distinctions difficult at best.

We believe that a more promising way forward, adopted in this and other recent studies, is the quantitative analysis of choice behavior in rigorously controlled behavioral contexts (for reviews see [26], [72], [73], [74], [75], [76], [77]). These studies attempt to encompass within a single quantitative framework (signal detection theory and its intellectual heirs) a rich array of well defined cognitive factors that influence choice, including but not restricted to sensory evidence, prior probabilities, reward value, and strategic interactions with competitors. These studies have the cardinal virtue of quantifying each proposed cognitive factor in terms of its impact on choice, and defining each factor in formal equations or in precisely specified simulations. Given these preconditions, scientific investigation can progress naturally by the development of new models that account demonstrably better for behavioral choice and the underlying neural signals. This approach conforms to Maunsell's [69] call for “accurate descriptions of behaviorally relevant information encoded in the brain,” and has the desirable outcome that terminology becomes less important to the field than quantitative understanding.

Perhaps the most general theory of LIP function that has come forth in recent years is that LIP contains a “salience map” or “priority map” of space that encodes the potential behavioral salience of each region of space [58], [78], [79]. Behavioral salience is proposed to arise from bottom-up processes such as the sudden appearance of a novel object [80], [81] and by *any* internal analysis of an object related to reward, arousal, choice, social significance (top-down)—in short anything that an animal might find significant either by natural inclination or by training [79]. Similarly, the output of the salience map can be used for *any* behavioral purpose including covert attention, guidance of eye or arm movements, etc. While intuitively congenial, the very generality of this idea renders it necessarily qualitative. Different behaviors will draw upon very different combinations of sensory input, behavioral goals, reward potential, and social significance to endow salience upon different regions of space. Thus quantitative understanding of LIP function, as suggested above, is more likely to arise from consideration of specific behavioral paradigms in which relevant inputs can be specified precisely, possible neural computations can be inferred from quantitative modeling of behavioral data, and neural signals can be compared to the internal computations postulated by the behavioral model [31], [73]. From this point of view the notion of a salience map is a useful heuristic for guiding inquiry, and most LIP data—including those presented in this paper—are broadly consistent with the notion of salience. But mechanistic understanding must ultimately emerge from more precise consideration of specific behaviors.
